# Aptamer-Based Biosensors for Antibiotic Detection: A Review

**DOI:** 10.3390/bios8020054

**Published:** 2018-06-11

**Authors:** Asol Mehlhorn, Parvaneh Rahimi, Yvonne Joseph

**Affiliations:** Institute of Electronic and Sensory Materials, Faculty of Materials Science and Materials Technology, Technological University Freiberg, Akademie Str. 6, 09599 Freiberg, Germany; Asol.Mehlhorn@esm.tu-freiberg.de (A.M.); yvonne.joseph@esm.tu-freiberg.de (Y.J.)

**Keywords:** biosensor, aptasensor, aptamer, antibiotic, ampicillin, penicillin, gentamicin, kanamycin, neomycin, tobramycin, streptomycin, daunomycin, chloramphenicol, ciprofloxacin, danofloxacin, enrofloxacin, ofloxacin, lincomycin, oxytetracycline, tetracycline, sulfadimethoxine

## Abstract

Antibiotic resistance and, accordingly, their pollution because of uncontrolled usage has emerged as a serious problem in recent years. Hence, there is an increased demand to develop robust, easy, and sensitive methods for rapid evaluation of antibiotics and their residues. Among different analytical methods, the aptamer-based biosensors (aptasensors) have attracted considerable attention because of good selectivity, specificity, and sensitivity. This review gives an overview about recently-developed aptasensors for antibiotic detection. The use of various aptamer assays to determine different groups of antibiotics, like β-lactams, aminoglycosides, anthracyclines, chloramphenicol, (fluoro)quinolones, lincosamide, tetracyclines, and sulfonamides are presented in this paper.

## 1. Introduction

The increase of antibiotic-resistant germs is an acute challenge for consumer health protection and veterinary medicine. Inappropriate and prophylactic use of antibiotics (especially in the field of animal care) is common and associated with contamination of the environment with antibiotics and their metabolites. On one hand, this favors the development of antibiotic resistances of bacteria, while, on the other hand, this harms the environment, e.g., by uncontrolled disturbance of the ground flora [1,2,3]. To minimize the resistance towards antibiotics the use and the release of them into the environment must be first detected and, thereupon, can be limited [4].

In their original sense antibiotics are naturally-built low molecular weight metabolites of bacteria or fungi, which either kill or slow the growth of other microorganisms [5]. In the widest sense, partial synthetic derivates and chemically-synthesized compounds with antimicrobial effect belong to antibiotics. They are generally used for treating infections in modern healthcare [5]. According to their chemical structure and the resulting mode of action, antibiotics can be classified into different groups inter alia ß-lactams, aminoglycosides, anthracyclines, (fluoro)quinolones, tetracyclines, lincosamide, and sulfonamides.

Currently, residue levels of antibiotics in aqueous samples are mainly detected by high-performance liquid chromatography (HPLC) [6,7,8,9], gas chromatography-mass spectrometry (GC-MS) [10], and liquid chromatography-tandem mass spectrometry (LC-MS/MS) [11,12,13,14]. Despite their wide range of applications, these methods are usually time-consuming, require laborious pretreatment of samples, sophisticated instrumentation, and trained technical personnel. The use of biosensors circumvent these problems and could ensure fast on-site analysis. Biosensors are analytical devices that contain two important functional components: a target recognition element (e.g., enzyme, protein, nucleic acid, or cell) and a signal transduction element [15]. According to their transducing element biosensors can be divided into mass-, optical-, and electrochemical-based biosensors [15,16]. Recently, several possible aptamer-based biosensors, known as aptasensors, have been developed for antibiotic detection. Aptamers are single-stranded DNA or RNA oligonucleotides, which can specifically bind to a wide range of target molecules, like nucleic acids, proteins, metal ions, and other small molecules with high affinity, selectivity, and sensitivity [17,18]. Due to such advantages in comparison to antibodies, aptamers are promising alternatives for most applications [19,20]. Suitable aptamers can be identified using a process called Systematic Evolution of Ligands by EXponential enrichment (SELEX) [17,18], first reported by Ellington et al. [17] and Tuerk and Gold [21]. In this approach, suitable binding sequences are first isolated from large oligonucleotide libraries and subsequently amplified. Aptamers can be produced at low cost and be easily modified with signal moieties [22]. Since the first publications of aptamer-based biosensors in 1996 by Drolet et al. [23] and Davis et al. [24] a variety of biosensors and assays have been successfully developed for aptamer-based analysis (recognition and detection) of different targets. Similar to the classical immunosorbent assays, aptamer assays can be designed as a single-site binding format, as a dual-site (sandwich) binding format, in which the analyte is sandwiched by a pair of aptamers, or a sandwich binding format with an aptamer and an antibody [25].

**Figure 1:**
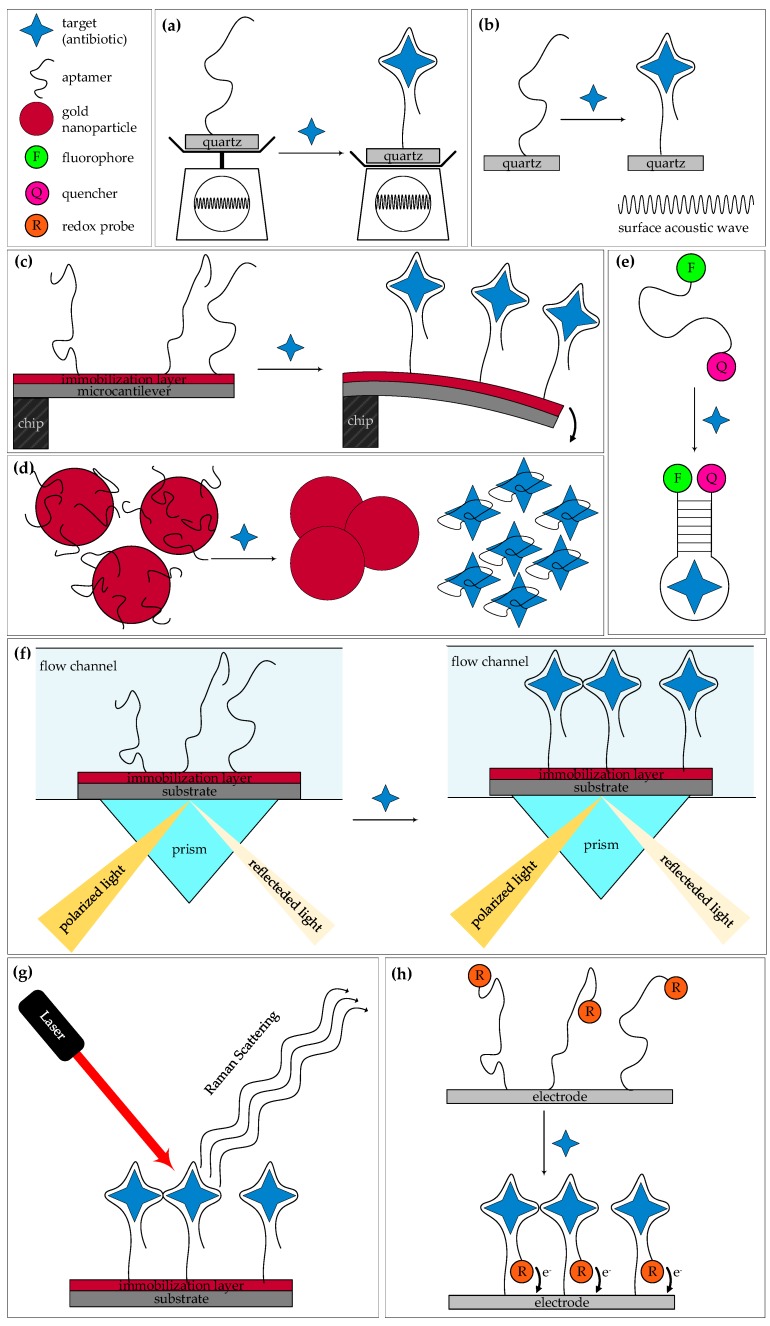
Working principles of the most widely used aptasensors. (**a**) Quartz crystal microbalance; (**b**) surface acoustic wave; (**c**) micromechanical cantilever array; (**d**) AuNPs based colorimetric aptasensor; (**e**) fluorometric aptasensor; (**f**) surface plasmon resonance; (**g**) surface enhanced Raman scattering; and (**h**) electrochemical aptasensor.

Aptasensors can be fabricated with various transducers that are mass-, optically-, or electrochemically sensitive. The corresponding transduction principles are given in Figure 1. In quartz crystal microbalance (QCM, Figure 1a) and surface acoustic wave sensors (SAW, (Figure 1b) the change of the oscillation frequency of an acoustic wave due to a target binding is measured [16,25]. In QCM the acoustic wave is produced as a bulk acoustic wave, while in SAW sensors the wave travels along the surface of an elastic material with an amplitude that typically decreases exponentially with the substrate depth [26]). In micromechanical cantilever arrays (MCA, Figure 1c) aptamer-target binding leads to a change in the resonance frequency of the microcantilever (dynamic mode) or to a steric crowding that forces the cantilever to bend (static mode). The bending is detected optically or electronically [16,25].

The most commonly used optical biosensors are based on colorimetric or fluorometric detection [27]. Colorimetry is the determination of the concentration of a substance in a (mostly) liquid phase by comparison with a color scale which, in turn, corresponds to a known concentration of the substance [28]. Colloid gold nanoparticles (AuNPs) have been broadly considered as a label for molecular sensing because of their diverse electronic and optical properties. They absorb and scatter light with high efficiency, are known as strong quenchers, and exhibit a wide range of colors [29]. Responsible for the colors is the phenomena of localized surface plasmon resonance (LSPR), in which the conducting electrons on the AuNPs surface collectively oscillate in resonance with incident light [30]. AuNPs possess a high surface free energy, good biocompatibility, and large surface area where molecules can be immobilized (e.g., aptamers), and are catalytically active [29,31]. In the gold nanoparticle-based colorimetric assay (CoA, Figure 1d) the aptamer is bound onto the surface of AuNPs and, thus, prevents their aggregation. Upon target binding the conformation of the aptamer changes from a random coil structure to a folded rigid structure; in consequence the adsorbed aptamers detach from the AuNPs and the AuNPs aggregate. This leads to a visible color change of the solution [27]. A limitation of AuNP-based colorimetric assays is the tendency of AuNPs to aggregate non-specifically in the presence of salt and other molecules present in the complex biological fluids [32]. In the fluorometric assay (FlA, Figure 1e) the aptamer is labelled with a fluorophore and an appropriate quencher. The binding of the target causes a conformational change of the aptamer and brings the fluorophore and quencher into close contact, whereupon the fluorescence is quenched [33]. This is known as “signal-off” mode. The reverse case, the “signal-on” mode, is possible too, whereby the conformational change upon target binding leads to a divergence of fluorophore and quencher, resulting in a fluorescence signal. Graphene oxide (GO) has been widely used as a fluorescence sensing platform because of its good biocompatibility, low cytotoxicity, and excellent capabilities for conjugation of target molecules [34]. GO and surface-modified graphene are highly efficient fluorescence quenchers based on either electron or energy transfer mechanisms [35,36]. Förster resonance energy transfer (FRET) is the mechanism of non-radiation (dipole-dipole) energy transfer from an excited chromophore (donor) to a second chromophore (acceptor) [37]. Upconversion nanoparticles (UCNPs) are nanoscale particles, exhibiting photon upconversion, which means that the sequential absorption of photons leads to the emission of light at shorter wavelengths than the excitation wavelength [38]. UCNPs possess a couple of advantages compared to other types of fluorescent materials, like organic dyes or fluorescent proteins, including higher photostability, low toxicity, large Stokes shifts, high quantum yields, and the lack of both auto-luminescence and a light-scattering background [39,40]. Quantum dots (QDs), semiconductor nanoparticles, belong to the UCNPs. Due to their influenceable optical and electronic properties, they are of interest for many applications and are applied as alternatives to molecular fluorophores in optical biosensors [41,42,43]. QDs are very small particles, with good conductivity, a high extinction coefficient, high chemical stability, broadband optical absorption, low toxicity and strong photoluminescence emission [44,45]. In contrast, the chemiluminescence resonance energy transfer (CRET) occurs by the oxidation of a chemiluminescent substrate without an excitation source [46]. The quantum mechanical phenomenon of the surface plasmon resonance (SPR, Figure 1f) is the fundamental principle behind many biosensor applications and different lab-on-a-chip systems utilized for detecting molecular interactions [25]. Polarized light, parallel to the incidence plane, strikes an electrically-conducting surface. Often thin metal films or semiconductor films, like gold, are used. At the interface between two media, a resonance interaction with oscillating electrons occurs, generating electron charge density waves, so-called surface plasmons which are totally reflected. When an analyte is bound, the refractive index of the film and, consequently, the resonance angle alters. Thus, the intensity of reflected light at a specific angle (known as the resonance angle) is changed, proportional to the mass on a sensor surface [28]. SPR is a versatile technique, in which no elaborate sample preparation and no radioactive or enzyme-labelled reagents are necessary [47]. The surface enhanced Raman scattering (SERS, Figure 1g) is a highly sensitive optical measurement method, that provides the signals based on the enhanced inelastic scattering of light on atoms or molecules (Raman scattering) immediate near a metal surface, often Au or Ag, with nanoscale roughness [48].

The principle of an aptamer-based electrochemical biosensor is the following (Figure 1h): the aptamer is immobilized onto an electrode surface and labelled with a redox probe (often ferrocene), methylene blue (MB), or Fe_3_O_4_ NPs). Upon target-binding the conformational change of the aptamer brings the probe closer to the electrode surface. An electron transfer, and thereby an electrochemical readout, is possible [27], which is known as “signal on” mode. Alternatively the conformational change increases the distance between the redox probe and the surface electrode, resulting in an interruption of the previous electron transfer, designated as “signal off” mode [25]. A simultaneous detection of more than one target analyte is possible by using various metal ions, e.g., Cd^2+^, Pb^2+^, Zn^2+^, and Cu^2+^, with diverse redox potentials to produce distinguishable electrochemical signals [49]. These kinds of probes are designated as metal-labelled biocodes [49]. Usually electrochemical measurements are carried out in a conventional three-electrode system, containing a working electrode (e.g., Au or glassy carbon), a reference electrode (e.g., Ag/AgCl or saturated calomel), an auxiliary electrode (e.g., platinum wire), and a redox probe in buffer solution (e.g., [Fe(CN)_6_]^4−/3−^). Four types of electrochemical sensors are distinguished by their measuring principle: (a) conductometric-based, which sense the change of electrical charge in a solution under constant voltage; (b) potentiometric-based, which sense changes in the electrical potential difference upon binding; (c) amperometric-based, which sense the difference in current potentials during redox reactions when pairing occurs; and (d) impedimetric-based, which sense changes in impedance upon interaction [16].

More methods for antibiotic detection based on the mentioned basic principles are described in detail in the appropriate sections of the paper. Additionally, further detailed information about the operating modes for aptamer-based biosensors can be read inter alia in the reported review papers [50].

This systematic and comprehensive review discusses the application of aptamers in the detection of different antibiotic groups. In this section, eight different groups of antibiotics, and the designed aptasensors for their detection, are discussed.

## 2. Aptasensors for Different Antibiotic Classes

The various currently-developed aptamer-based biosensors for antibiotic detection mentioned in the literature are ordered by their antibiotic class and discussed below.

To compare the performance of aptasensors the following parameters or characteristics are important.

Affinity is a measure of the tendency of molecules to bind to other molecules [51]. The higher the affinity, the greater the association constant K_A_. More common is the reciprocal value, the dissociation constant K_D_. The higher the affinity of a target to its ligand, the lower the K_D_ of the complex, thus, low K_D_ values are preferred.

Selectivity is the property to select multiple objects from a set of objects, while specificity is the property to select one object from a set of objects [52]. Thus, an analytical method is selective when different components of a mixture can be determined side by side and without interference. The method is specific when only one component of the mixture can be determined. Specificity tests are usually carried out by target detection in the simultaneous presence of the target and structurally similar substances. High specificity and selectivity is preferred.

The limit of detection (LOD) is the lowest quantity of a substance that can be distinguished from the absence of that substance (a blank value) within a stated confidence limit [53]. The maximum residue levels (MRL) is the highest concentration of an undesirable substance (impurity or pollutant), that is legally permitted in a food or commodity [54], defined by the European Union, e.g., in Council Directive 96/23/EC [54] for antibiotic residues in live animals and animal products. The aim in the development of a biosensor is to achieve a low sensitivity, such that the LOD is smaller than the MRL.

The reproducibility is the repeatability of scientific research results [55].

The recovery is determined by a standard addition method. Defined target concentrations are added to real samples and the recovery is detected. Furthermore, the results are compared to results with an alternative method, the enzyme-linked immunosorbent assay (ELISA). 

The applicability of the proposed aptasensor for real-sample analysis is verified by the detection of the target in real samples, such as milk, honey, serum, water, and others. 

The stability is the ability of the sensor to maintain its performance under the prevailing conditions for a certain period of time. It is tested by storing the sensor at defined conditions up to several weeks, comparing the analytical performance before and after storage.

### 2.1. β-Lactams

**Figure 2:**
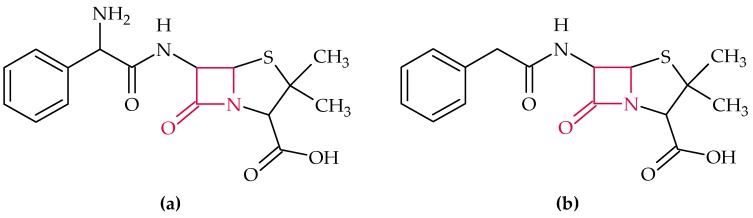
Chemical structure of: (**a**) ampicillin and (**b**) penicillin G. The β-lactam ring is marked in red.

Due to their high efficacy, low toxicity, and the possibility to derivatize them by means of chemical and enzymatic methods, β-lactam antibiotics are considered to be the most important antibiotics in terms of quantity and value [56]. Their mechanism of action is based on the prevention of the formation of peptide cross-linking in the bacterial cell wall (murein) [5]. Therefore, they act specifically on prokaryotes with a mureous cell wall. They are characterized by their representative β-lactam ring (marked in red in Figure 2). The most important data of the discussed aptasensors, for the β-lactams, include the aptamer sequence, dissociation constant (K_D_), limit of detection (LOD), real sample analysis (RSA), and applied sensor type, are summyarized in Table 1 and Table 2.

#### 2.1.1. Ampicillin

The occurrence of penicillin-resistant strains has stimulated the search for new antibiotics, from which semi-synthetic penicillin, such as ampicillin, have been found [57]. Ampicillin (Figure 2a) is a widely used broad spectrum antibiotic in veterinary medicine for the treatment and prevention of primary respiratory, gastrointestinal, urogenital, and skin bacterial infections in food-producing animal and it has a low human toxicity [57].

Song et al. [58] reported the first aptasensor for ampicillin using AuNP-based dual fluorescence colorimetric methods. Ampicillin-specific aptamers were selected by magnetic bead-based SELEX, which was further used in other studies [59]. Another fluorescent aptasensor for ampicillin detection was reported by Luo et al. [60] using magnetic bead composites coated with AuNPs and a nicking enzyme, which showed more sensitive responses with the limit of detection of 0.07 ng mL^−1^ compared to the first proposed aptasensor by Song et al. 

Dapra et al. [59] designed an all-polymer impedimetric electrochemical microfluidic biosensor for the detection of ampicillin and kanamycin A. Cyclic olefin copolymer (Topas^®^) was used as a substrate on the top and bottom of the constructed chip. Due to their biocompatibility a conductive polymer bilayer consisting of tosylate-doped poly(3,4-ethylenedioxythiophene) (PEDOT) and the hydroxymethyl derivate was used as the electrode material. In a similar system Rosati et al. [61] optimized the geometry of the electrodes and the microchannels (e.g., thickness, width) and, therefore, the performance of the designed impedimetric aptasensor. Additionally, the ampicillin-specific aptamer was equipped with an appended poly(T)-poly(C) sequence which allowed a direct immobilization on the electrodes when UV irradiation was applied [62].

In order to enhance the aptasensor response to ampicillin and signal amplification, two electrochemical aptasensors were developed with the help of polymerase and nicking endonuclease [63], which was used for the first time as the target-aptamer binding triggered quadratic recycling amplification for electrochemical detection of antibiotics, and target-initiated T7 exonuclease in a homogeneous electrochemical sensing system [64].

There are many reports on electrochemical-based aptamer sensing assays for the detection of ampicillin [63,64,65,66,67,68], which are summarized in Table 1. Among all existing electrochemical sensor assays, the proposed aptasensor based on DNA-functionalized AuNPs and ssDNA binding protein (SSB) as the electrochemical signal inhibition reagent showed higher sensitivity with a detection limit of 0.38 pM [65]. 

#### 2.1.2. Penicillin

Penicillin G (Figure 2b) is used for the production of 6-aminopenicillanic acid (6-APA), the main intermediate product for the synthesis of semi-synthetic penicillins and cephalosporins [56]. It is the most frequently used β-lactam antibiotic for the prevention and treatment of bacterial infections, like scarlet fever, diphtheria, gonorrhoea, angina, and tetanus. Penicillin is produced by the fungi *Penicillium notatum* and is hardly humanly toxic [57].

The first aptasensor for detection of penicillin was reported by Zhao et al. [69]. They developed an electrochemical aptasensor using a composite film consisting of a magnetic graphene nanocomposite (GR–Fe_3_O_4_NPs) and a poly(3,4-ethylenedioxythiophene)–gold nanoparticle composite (PEDOT–AuNPs) for the modification of the electrode to assemble the penicillin aptamer to it. 

Paniel at al. [70] described the selection of aptamers selective to penicillin G using the capture-SELEX process. The process is based on the selection of DNA aptamers using the ssDNA fixed on a support, whereas the target is in solution. Selectivity tests showed that the aptamer was able to bind other β-lactam antibiotics, including amoxicillin and ampicillin, indeed with less affinity.

Lee et al. [71] identified ssDNA aptamers for the detection of penicillin G by reduced graphene oxide-SELEX (rGO-SELEX). rGO-SELEX is a method which uses the π-π stacking interaction between rGO and nucleic acids for an immobilization-free selection of aptamers. Furthermore, rGO is an effective fluorescence quencher through the FRET effect. Thus, the fluorescence recovery signal from the quenched FAM-labelled aptamer on the rGO surface can give information about the binding of a target to the aptamer.

The comparison between the different aptasensors for penicillin G are shown in Table 2. Although penicillin is an important and widely used antibiotic, only a few aptamer-based biosensors exist for its detection. Hence, there is still potential for research.

### 2.2. Aminoglycosides

**Figure 3:**
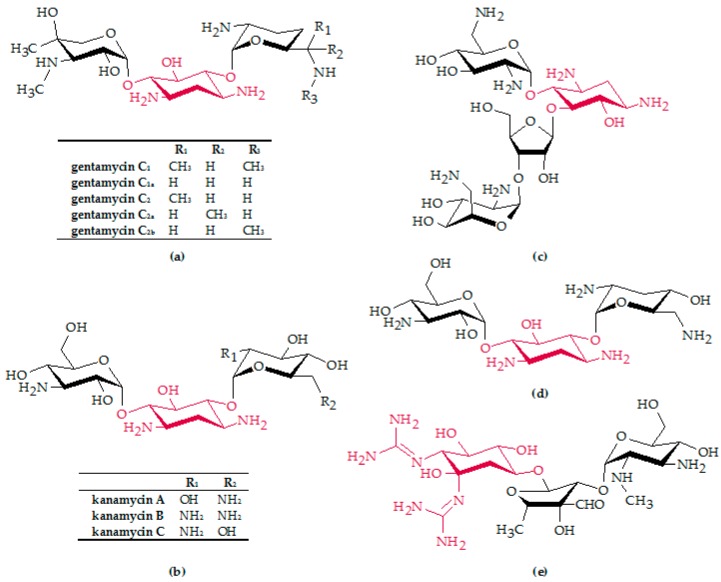
Chemical structure of: (**a**) gentamicin; (**b**) kanamycin; (**c**) neomycin B; (**d**) tobramycin; and (**e**) streptomycin. The basic structure of aminoglycoside antibiotics consists of an aminocyclitol ring (marked in red) which is linked glycosidically to other amino sugars.

Aminoglycoside antibiotics are the most commonly used antibiotics worldwide, with a broad spectrum of activity—also against Gram-negative bacteria [56]. Despite their relatively high toxicity (especially on the ears and kidneys), they are the antibiotics of severe infections which, in turn, leads to an increase in resistance [56]. They exert their effect by binding to the 30S subunit of ribosomes, which leads to reading errors during translation and, ultimately, inhibition of protein biosynthesis [56]. The basic structure of most aminoglycoside antibiotics consists of an aminocyclitol ring which is linked glycosidically to other amino sugars (Figure 3) [57]. The most important data of the discussed aptasensors, for the aminoglycosides including aptamer sequence, K_D_, LOD, RSA, and applied sensor type, are summarized in Table 3, Table 4, Table 5, Table 6 and Table 7.

#### 2.2.1. Gentamicin

The alkaline aminoglycoside antibiotic gentamicin (Figure 3a), isolated in 1963, is a broad-spectrum antibiotic and acts as a bactericide against a large number of Gram-positive and Gram-negative pathogens, such as *E. coli* and *Pseudomonas* [56]. It is used especially for severe wound infections and accidental injuries, as well as secondary infections after burns [56]. Therapeutically-used gentamicin usually contains 70% of the C_1_ and 30% of the C_2_ component (Figure 3a) [56]. Gentamicin is less toxic than kanamycin, neomycin, and streptomycin. However, ototoxic and nephrotoxic damage have been observed [56].

Rowe et al. [72] designed an electrochemical aptasensor based on RNA-aptamers and DNA-aptamers for the detection of the aminoglycoside antibiotics gentamicin, tobramycin, and kanamycin in blood samples in order to prevent overdosage and side effects. DNA aptamers were more stable, but the SWV experiments showed a lower sensitivity of the DNA-aptamer-based sensor to detect aminoglycosides in blood samples compared to the RNA-based sensor. The most important data of the developed sensor given in Table 3.

This work is the only aptasensor for gentamicin detection studied up to now in the literature, therefore, great potential exists for further research.

#### 2.2.2. Kanamycin

Kanamycin (Figure 3b) is a bactericidal antibiotic isolated from *Streptomyces kanamyceticus*, whose spectrum of activity comprises Gram-positive and Gram-negative bacteria [56]. It is widely used as a veterinary drug and as a second-line antibiotic to treat serious infections, such as pneumonia, septicemia, urinary tract infections, and intestinal infections [72]. If not explained in more detail, kanamycin stands for a mixture of kanamycin A, B, and C, in which kanamycin A has the largest share [57]. Since sensitive and selective methods to detect kanamycin residues for food safety and clinical diagnosis are of great interest, there are more reports in kanamycin aptamer-based sensors in comparison to other antibiotics.

Song et al. [74] discovered the kanamycin-specific aptamer, which was later used in a variety of other studies [75] (Table 4). They selected the aptamer in vitro by SELEX using affinity chromatography with kanamycin-immobilized sepharose beads. The specific aptamers were immobilized onto the AuNPs to fabricate a colorimetric-based aptasensor. In the presence of kanamycin, the addition of salt leads to an aggregation of the modified AuNPs, which results in a color change from red to purple. 

There are several reports for kanamycin detection using different colorimetric-based biosensing assays, due to its low cost, simplicity, and observation of the color change by the naked eye [32,74,75,76,77,78,79]. However, among them, silver nanoparticles (AgNPs) and AuNP-based colorimetric aptasensors have attracted more and more attention due to size/distance-dependent optical properties [76,77]. Thus, different research groups have tried to design a sensitive and specific kanamycin aptasensor based on AgNPs and AuNPs for the possible application in food or clinical samples [76,77,78,79].

Wang et al. [80] designed a colorimetric aptasensor for the detection of kanamycin based on liquid crystal film. Amino-functionalized kanamycin-specific aptamers and *N*,*N-*dimethyl-*N*-(3-(trimethoxysilyl)propyl)-1-octadecanaminiuchloride (DMOAP) were co-immobilized onto the surface of a glass slide, resulting in a homeotropic orientation of the liquid crystal film. The addition of kanamycin resulted in the formation of G-quadruplex structures, which destroyed the oriented arrangement of the liquid crystals on the surface and caused a visible color change from pink to green.

Fluorescence, as one of the most common optical techniques, has been used in the fabrication of aptasensors for kanamycin detection [81,82,83,84,85,86,87,88,89,90]. Generally, the binding affinity of an aptamer towards its target decreases by tagging the aptamer with a fluorescent dye [83]. Therefore, it is needed to improve the sensitivity of the designed aptasensors. Regarding this fact, different strategies have been employed for amplification of fluorometric signals. Nanoparticles (NPs), due to their advantages, can be used as the modifier films and improve the sensitivity of fluorometric aptasensors. Different NPs have been employed in the construction of highly sensitive fluorometric aptasensors, like silica NP (SNPs) [81], amino-Fe_3_O_4_ magnetic NPs [82], AuNPs [84], and UCNPs [86]. Immobilization of aptamers on the surface of reduced graphene oxide (rGO) [88] and carbon nanotubes (CNTs) [89] have showed great promise in the amplification of fluorometric signals in the detection of kanamycin. Moreover, Wang et al. [87] developed a fluorometric aptasensor, based on MoS_2_ nanosheets and carbon dots. The quenching ability of layered MoS_2_ was tested and compared to that of GO and AuNPs and showed comparable, or even better, values. 

Chemiluminescence-based aptasensor in comparison with other optical aptasensors, like fluorometric and colorimetric aptasensors, has the lowest LOD [88]. However, there are a few reports in aptamer-based luminescence methods for the detection of kanamycin [91,92,93], which are summarized in Table 4. 

Electrochemical aptasensors compared to optical sensors are label-free, simples, more practical and sensitive, and have attained a great deal of attention in the detection of antibiotics [72]. In order to enhance the specificity and sensitivity of the designed electrochemical aptasensors, the surface of electrodes were modified with different functional groups, nanomaterials, polymers, or nanocomposites to immobilize aptamers or standard targets. 

An impedimetric disposable and portable aptasensor for the detection of kanamycin was designed by Sharma et al. [99]. Amino-functionalized kanamycin-specific aptamers were immobilized onto the surface of the working screen-printed carbon electrode (SPCE) via NH_2_-COOH interaction. The interaction between the aptamers and kanamycin caused an inhibition in the Faradaic response and an increase in the electron transfer resistance. 

The first label-free electrochemical biosensor for kanamycin detection based on an aptamer-functionalized conducting polymer-Au nanocomposite modified disposable screen-printed electrode (SPE) was reported by Zhu et al. [100]. Many other researchers have tried to design the sensitive electrochemical sensing system for kanamycin detection. For example, Sun et al. [101] introduced an electrochemical sensor based on synergistic contributions of different nanocomposites, including chitosan-AuNPs (C-AuNPs), graphene-AuNPs (G-AuNPs), and multi-walled carbon nanotube (MWCNT)-cobalt phthalocyanine composites (MWCNT-CoPc) in order to enhance the electron transfer processes and the response speed of the aptasensor. Several nanocomposite films, e.g., graphene polyaniline/AuNPs [102,104], CNTs/ionic liquid/nanoporous platinum titanium alloy [105], CNTs/IL/graphene [106], nanoporous PtCu/graphene [107], and metal ion-doped nanoscale metal organic frameworks (MOFs) [111], have been used by other groups to improve the aptamer immobilization and fabricate a sensitive aptasensor for kanamycin. 

Sometimes, in order to obtain high specificity and improve the sensitivity of aptasensors, the biocatalytic properties of enzymes are used to detect and amplify the analysis of targets with their aptamers. For example, horseradish peroxidase (HRP) [108] or glucose oxidase (GlO) [113], conjugated with AuNP-cDNA, have been used as biocatalysts for signal amplification for the detection of kanamycin. 

Photoelectrochemical sensing is a novel method with high sensitivity and rapid response which combines the benefits of optical methods and electrochemical sensors [83]. Li et al. [96] developed a photoelectrochemical aptasensor for the detection of kanamycin based on the graphene-modified flour-doped SnO_2_ electrode. In the absence of kanamycin, the generated photocurrent was low. When kanamycin was added, it was trapped by the aptamers on the surface of the electrode. The captured molecules were oxidized by photogenerated holes. The recombination of photogenerated holes and electrons was inhibited, resulting in an amplified photocurrent. 

In other reports, AuNPs-functionalized self-doped TiO_2_ nanotube arrays [97] and polypyrrole/CeO_2_/AuNPs [98] have been used as the photoactive materials to fabricate the photoelectrochemical aptasensors for kanamycin detection.

Several research efforts have been also made toward the design of aptasensors for detection of kanamycin A based on fluorometric [114,115] and electrochemical methods [59,116]. Nikolaus and Strehlitz [113] selected DNA-aptamers specific for binding of kanamycin A by capture SELEX according to the work of Stoltenburg’s team [117] and further tested in bead-based or microplate-based assays by fluorescence detection of the 5′-FAM-labelled aptamers. By the way, Robati et al. [88] authored a review about aptasensors for quantitative detection of kanamycin and kanamycin A. 

In summary, around half of all developed aptamer-based biosensors for the detection of kanamycin and kanamycin A are based on electrochemical sensor principles (either impedimetric or amperometric). Moreover, a comparatively large number of fluorometric aptasensors have been developed. The most important data of the discussed aptasensors are summarized in Table 4. The lowest LOD was reached with an amperometric aptasensor developed by Wang’s team [110].

#### 2.2.3. Neomycin

The spectrum of activity of neomycin (Figure 3c) is mainly Gram-negative bacteria, including *Salmonella* and *Shigella*. Since it is hardly absorbed after oral administration, it is particularly suitable for combating infections of the digestive tract. It is also used for superficial skin and mucous membrane infections. A disadvantage is the high ear and kidney toxicity. In general, neomycin is an oligosaccharide mixture containing the three main components A, B, and C. Commercially available neomycin consist of about 90% neomycin B and 10% neomycin A and B [57].

In 1995 Wallis et al. [118] selected RNA-aptamers for neomycin B recognition by in vitro selection using SELEX, which was used in further studies to fabricate a fluorometric aptasensor based on AuNPs [119] and an impedimetric electrochemical aptasensor based on the immobilization of aptamers on the surface of a modified electrode with self-assembled monolayer (SAM) of mercaptopropionic acid [120].

In 2009 de-los-Santos-Alvarez et al. [121] studied how the modification of the RNA-aptamer influences the affinity of the interaction between the aptamer and neomycin B. In general, the fully 2′-*O*-methylization of the RNA-aptamer should prevent the degradation by endonuclease. They showed that this modification did not significantly alter the aptamer affinity towards neomycin B, but the proposed aptasensor was more sensitive towards neomycin B in comparison with other aptasensors (Table 5).

The comparison of the obtained LODs (Table 5) showed that SPR [121] is more sensitive than the optical [119] and the electrochemical [120] method.

#### 2.2.4. Tobramycin

Tobramycin (Figure 3d) is a semi-synthetic aminoglycoside antibiotic [56]. Its spectrum of activity comprises numerous Gram-negative pathogens, such as *Escherichia coli*, *Klebsiella*, *Proteus*, *Pseudomonas*, *Salmonella*, and *Shigella*, as well as Gram-positive *Staphylococci* and *Enterococci* [57]. It is therapeutically effective for infections of the respiratory and the urogenital tract, the skin, bones, the central nervous system (meningitis), and septicemia [57].

In 1995 Wang and Rando [73] selected RNA molecules that could specifically bind to the aminoglycoside antibiotic tobramycin by in vitro selection using SELEX and used in later studies [72].

Spiga et al. [122] introduced a DNA-based capture-SELEX coupled with in-stream direct-specificity monitoring via SPR. The aptamers were evaluated for their affinity to tobramycin via direct immobilization onto a SPR chip, which was used in further studies [123].

Han et al. [124] developed a magnetic bead-based SELEX to identify 37 ssDNA aptamers specific for tobramycin using a fluorescent method based on the reported principle by Ma’s team [125], in which they developed a colorimetric aptasensor for the determination of tobramycin in milk and chicken eggs based on the adsorption of ssDNA aptamers on the surface of AuNPs. With the sensor of Ma et al. [125] the one reaching lowest LOD was developed (Table 6).

In order to detect drug concentration in patient samples, which are much more complex matrices than buffers, Cappi et al. [123] developed a portable, palm-sized transmission-localized SPR (TL-SPR) system for tobramycin detection. They used a setup based on aptamer-functionalized gold nanoislands (NIs) deposited on a glass slide covered with fluorine-doped tin oxide (FTO), which acts as a biosensor, and a complementary metal oxide semiconductor (CMOS) as a light detector. The sensitivity of the CMOS image sensor was matched to the localized plasmon resonance exhibited by the Au-NIs. For the first time it was shown that label-free direct detection and quantification of a small molecule can be reliably used in the complex matrix of filtered undiluted blood serum.

In an effort Gonzalez-Fernandez et al. [126] evaluated and compared the affinity and analytical characteristics of two partially and fully O-methylated modified RNA-aptamers for the design of electrochemical aptasensors for tobramycin detection in human serum. In addition to the higher endonuclease resistance, the fully O-methylated aptamer had a lower dissociation constant, as well as a lower LOD than the partially-methylated aptamer (Table 6), which was used in further experiments to develop the aptamer-based inhibition assays for detection of tobramycin [127,128]. 

Schoukroun-Barnes et al. [128] presented a systematic study of several approaches to develop an electrochemical RNA aptamer-based biosensor for the detection of aminoglycoside antibiotics, like tobramycin. They could design a highly sensitive aptasensor for tobramycin through the optimization of the electrochemical interrogation parameters and biomolecular engineering of the RNA aptamer-sequence (Table 6).

In summary, there are just a few aptasensors developed for tobramycin detection and with the exception of one, they are based on electrochemical principles. Almost all electrochemical sensors used RNA aptamer sequences for the specific tobramycin recognition. The loweset LOD and belonging K_D_ value was determined with the RNA aptamer sequence II (Table 6), mentioned by Schoukroun-Barnes et al. [128]. An even higher affinity towards tobramycin was reached by Cappi et al. [123] by using ssDNA aptamer sequence (Table 6).

#### 2.2.5. Streptomycin

The discovery of streptomycin (Figure 3e) from *Streptomyces griseus* by Selmon Waksman (1943) allowed, for the first time, a therapy of the tuberculosis pathogen *Mycobacterium tuberculosis*. However, due to the renal and ear-harming properties of streptomycin, other antibiotics (e.g., rifampicin) are usually used today [56]. More frequently, it is used to combat penicillin-resistant strains of *Neisseria gonorrhea* infections and still used for the treatment of tuberculosis [57].

The first streptomycin-specific DNA-aptamers was screened by Zhou et al. [130] by affinity magnetic bead-based SELEX. Streptomycin was detected by using a label-free AuNP-based colorimetric method.

Liu et al. [131] developed an aptamer-based colorimetric sensor for the detection of streptomycin. Different streptomycin-specific aptamer sequences were obtained by SELEX. The selected aptamer was used for all further experiments. 

Based on the interesting features of AuNPs for the construction of colorimetric aptasensor, there are several reports of such biosensors for streptomycin detection [130,131,132,133,134].

A colorimetric and fluorescence quenching aptasensor for streptomycin detection, based on the specific aptamer and its FAM-labelled complementary strand (cDNA) and aqueous AuNPs was reported by Emrani et al. [134]. Comparisons between the pure colorimetric and the pure fluorometric method showed higher sensitivity of the aptasensor by measuring with the fluorometric one. Consequently, a variety of studies have been carried out to fabricate the high-sensitivity aptasensors for streptomycin based on the fluorometric method [135,136,137].

Xu et al. [136] developed a photoelectrochemical aptasensor for streptomycin detection based on CdTe QDs single-walled carbon nanohorns, synthesized via the one-pot method, which acted as the photoactive species. These could inhibit electron-hole pair recombination, accelerate electron transfer, and improve the photocurrent intensity. 

Ghanbari and Roushani [137] introduced an impedimetric electrochemical aptasensor for the detection of streptomycin based on the immobilization of streptomycin aptamers on the surface of a graphene QDs/AuNP nanocomposite. Upon addition of streptomycin, aptamer-target complexes were formed, causing an increase of the electrochemical signal. 

Yin et al. [138,139,140] constructed three quite similar electrochemical aptasensors for the detection of streptomycin based on the immobilization of the aptamer on the surface of modified electrodes with different nanocomposites, including MWCNTs/copper oxide (CuO)/AuNPs [138], AuNPs/magnetic MWCNTs/nanoporous PtTi alloy [139], and graphene/Fe_3_O_4_/AuNPs [140]. Comparisons of the current responses of the aptasensors to streptomycin and to a mixture of streptomycin and interfering substances confirmed an excellent specificity and high sensitivity of the sensor based on an AuNPs/magnetic MWCNTs/nanoporous PtTi alloy modified electrode towards streptomycin.

Summarized, there are only a few papers dealing with aptasensing of streptomycin. According to the data of Table 7, more than half of them are are based on electrochemical measurements. The up to five orders of magnitude lower LOD than those reached with the other sensors, could be determined by Luan et al. [132] using a colorimetric assay and was followed by Yin et al. [139] with an amperometric aptasensor.

### 2.3. Anthracyclines

**Figure 4:**
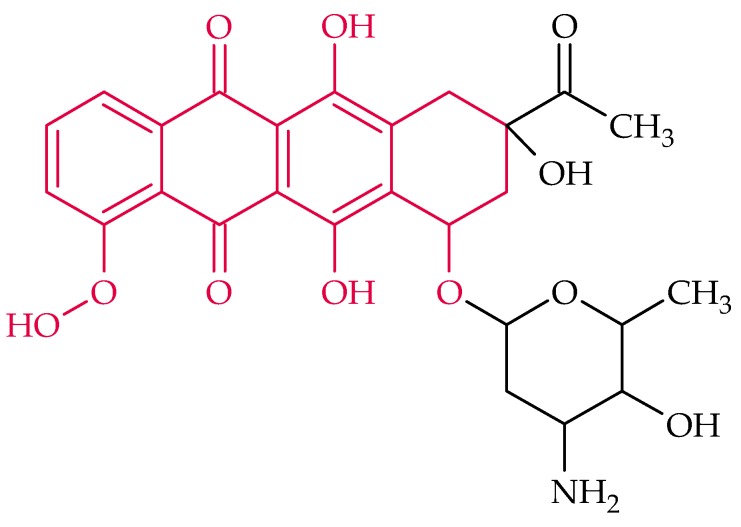
Chemical structure of daunomycin. The basic structure of the anthracyclines is marked in red.

Anthracyclines inhibit the replication of DNA by intercalation and inhibition of topoisomerases. They are used clinically for the treatment of tumors, but they can cause heart damage in the long-term medication [144]]. The basic structure, which all anthracyclines exhibit, is marked in red in Figure 4. 

#### Daunomycin

Daunomycin (Figure 4), the first discovered anthracycline, produced naturally by *Streptomyces peucetius*, acts as an intercalator whereat the intercalation between DNA bases leads to a local structural change in the DNA and, thus, to an inhibition of DNA replication and transcription [145]. Therefore, daunomycin has a growth inhibitory effect on Gram-positive bacteria and fungi. Moreover, an antiviral effect by inhibiting viral DNA replication in the host cell was obtained. In 1963 an antileukemic activity was discovered [57]. Nowadays daunomycin is widely used for the treatment of breast tumors, lymphocytic and myeloid leukemia [146].

In 2008 Wochner et al. [147] selected ssDNA aptamers, specific for daunomycin and tetracycline, which were used in further studies for the fabrication of an aptasensor [148]. 

In the work of He et al. [146] a colorimetric aptasensor for daunomycin detection based on resonance scattering is described. A fluorescence spectrophotometer was used to record the resonance scattering intensity. 

Chandra et al. [148] developed an electrochemical biosensor for daunomycin using the co-immobilization of the specific aptamers and phosphatidylserine on Au nanoparticle-deposited conducting polymer, which exhibited a higher sensitivity than the others (Table 8). 

### 2.4. Chloramphenicol

**Figure 5:**
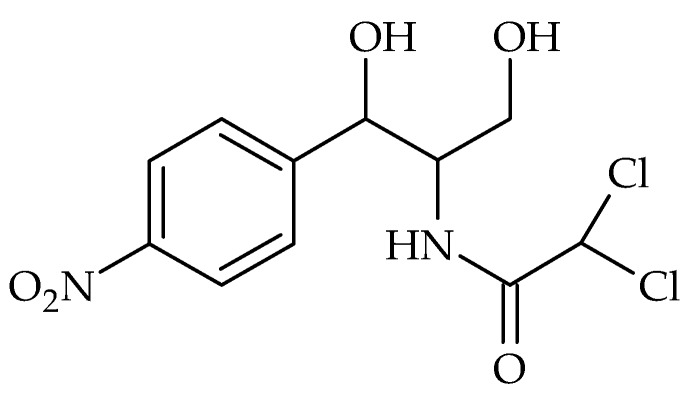
Chemical structure of chloramphenicol.

Chloramphenicol is an antibiotic class of its own [56]. The chemical structure is shown in Figure 5 [57]. It blocks the peptidyl transferase by binding to the 50S subunits of the 70S ribosomes [56]. It was isolated in 1950 from *Streptomyces venezuelae*, but nowadays it is exclusively produced synthetically [56]. It acts against Gram-positive and Gram-negative pathogens, as well as against *Actinomycetes*, *Rickettsiae*, and some large viruses [57]. Due to its serious side effects, such as leukemia, aplastic anemia, and grey baby syndrome, it is only a reserve antibiotic used to treat typhoid, shigellosis, and rickettsial infections [57,149].

In 2011 chloramphenicol-specific aptamers were selected and characterized by Mehta et al. [150] using the SELEX procedure, which was used in further studies for chloramphenicol detection [151,152].

Miao and colleges developed seven different aptasensing strategies for chloramphenicol detection [42,43,152,153,154,155,156]. Two aptasensors based on a colorimetric [152,153], one of them using electrochemiluminescence [154] and four aptasensors based on fluorometric principles [42,43,155,156].

In order to amplify the signals of colorimetric aptasensors, Miao’s team utilized the enzyme-linked polymer nanotracers labeled by a double-stranded DNA (ds-DNA) antibody. The aptamer was immobilized on Fe_3_O_4_/Au magnetic nanoparticles as a capture probe, and an enzyme-linked polymer nanotracer was fabricated by co-immobilization of HRP-labelled AuNPs and double stranded DNA (dsDNA) antibodies as signal tags on EnVision reagent, a kit containing about 100 HRPs and some anti-IgG [152]. The proposed aptasensor showed to sensitively respond down to 0.015 ng mL^−1^ towards chloramphenicol.

Based on magnetic aptamer-enzyme co-immobilization platinum nanoprobes and exonuclease-assisted target recycling, Miao’s team [153] designed a triple amplification colorimetric aptasensor with a detection limit of 0.3 pg mL^−1^ towards chloramphenicol. 

There are several reports in fluorometric aptasensors for chloramphenicol detection based on the immobilization of aptamers on different composites and using various prepared capture and signal probes [42,43,155,156,157,158,159,160]. Their analytical features are summarized in Table 9. As seen, the proposed “off-on” fluorometric aptasensor by Miao et al. [42] using vesicle QD-Au colloid composite probes, showed higher sensitivity towards chloramphenicol. They used the vesicle nanotracer as a signal probe, consisting of liposome-CdSe/ZnS QD complex labelled with SSB. Aptamer-functionalized AuNPs acted as the capture probe. The composite probe does not emit fluorescence signals, which represented the “off” state. Upon addition of chloramphenicol, the aptamer bound to it and the aptamer-target complex detached from the composite probe. The result is a fluorescence signal, which represents the “on” state. 

Based on the sensor principle to develop a electrochemiluminescent aptasensor for the detection of chloramphenicol [149,161,162], a triple-amplification assay using polymer enzyme-linked nanotracers/Exonuclease-assisted target recycling method [154] and TiO_2_-based nanorod assay sensitized with Eu(III)-doped CdS QDs as the photoactive material [163] were designed with a detection limit of 0.034 and 0.36 pM towards chloramphenicol, respectively.

Like the other antibiotics, there are more reports of constructed electrochemical aptasensors for the detection of chloramphenicol [41,49,112,164,165,166,167,168,169,170,171,172,173,174,175] As shown in Table 9, the lowest LOD could be obtained using the proposed electrochemical aptasensor based on Y-shaped DNA probes [174]. These probe-based metal ions encoded the nanoscale metal-organic frameworks (NMOF) as a substrate, and a circular strand-replacement DNA polymerization (CSRP) target triggered the amplification strategy. The proposed strategy exhibited a high sensitivity to chloramphenicol with a detection limit of 33 fM.

### 2.5. (Fluoro)Quinolones

**Figure 6:**
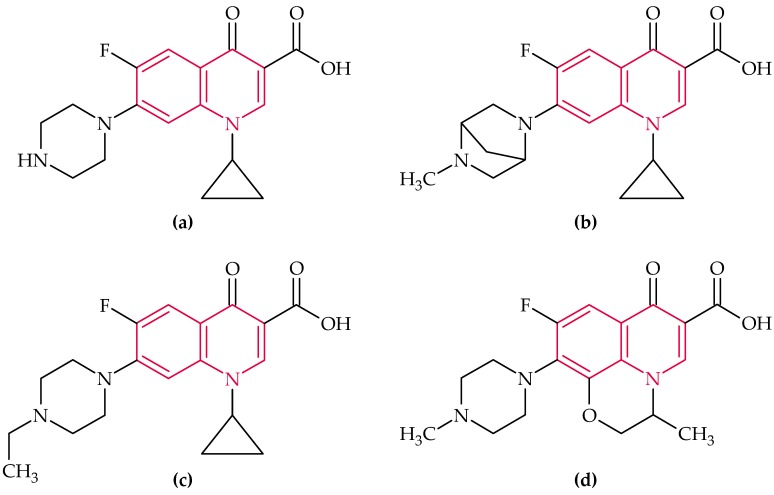
Chemical structure of: (**a**) ciprofloxacin; (**b**) danofloxacin; (**c**) enrofloxacin; and (**d**) ofloxacin. The structure of quinolone is marked in red.

(Fluoro)Quinolones have a very broad spectrum of action, thus, they act against Gram-positives, Gram-negatives, *Mycobacteria*, *Chlamydia*, and anaerobes, and are just slightly toxic to humans. Their mechanism of action based on the inhibition of DNA-gyrase, which belongs to the group of topoisomerases II. Inter alia, DNA-gyrase is responsible for the derivatization of the DNA. Structurally, quinolones are derived from quinolone (marked in red in Figure 6) [56]. The efficacy of the quinolones was further enhanced by the introduction of an additional fluorine atom, resulting in a whole series of fluoroquinolones.

#### 2.5.1. Ciprofloxacin

Ciprofloxacin (Figure 6a), a second-generation fluoroquinolone, which acts against *Bacillus anthracis*, the causative agent of anthrax, and is one of the most used quinolones nowadays [5,56].

There are just two reports of aptasensors for ciprofloxacin detection. In 2017 Lavee et al. [179] developed for the first time a colorimetric aptamer-based assay for the determination of ciprofloxacin using AuNPs. In another study an electrochemical aptasensor for ultrasensitive detection of fluoroquinolones, especially ciprofloxacin, based on a single-stranded DNA-binding protein, was presented [180].

The electrochemical aptasensor [180] possess 1.5-fold lower LOD than the colorimetric one [179] (Table 10).

#### 2.5.2. Danofloxacin

Danofloxacin (Figure 6b) acts against Gram-positive and Gram-negative bacteria and is often used for the treatment of respiratory diseases of cattle and pigs [181]. It is exclusively used in animal husbandry, not least because of its toxicity to humans [181]. 

By the application of SELEX, Han et al. [181] selected specific and high-affinity RNA aptamers with 2′-fluoro-2′-deoxyribonucleotide-modified pyrimidine nucleotides bound to danofloxacin. As a consequence, they employed an optical aptasensor for the detection of danofloxacin in buffer. The most important data of the discussed aptasensor are given in Table 11.

There are no other reports about investigations for danofloxacin detection by an aptasensor mentioned in the literature. 

#### 2.5.3. Enrofloxacin

Enrofloxacin (Figure 6c) is a high-potency antibacterial agent which is widely employed for disease prevention and therapy in poultry and livestock breeding and aquaculture practice [182,183].

For the detection of enrofloxacin, Liu et al. [182] designed a fluorometric aptasensor based on the immobilization of aptamers on the surface of Yb, Er ion-pair doped magnetic Fe_3_O_4_ UCNPs and amino-functionalized silica-modified (NH_2_-Si) UCNPs.

Moreover, Liu’s group [183] developed a fluorometric “double recognition” aptasensor for the detection of enrofloxacin by integrating two antibiotic recognition elements, including aptamers and fully-synthetic molecularly-imprinted polymers (MIPs) The LOD of the proposed aptasensor was about five times lower than the previously presented “simple” one [182] (Table 12), which is presumably related to the improved recognition ability of the sensor by the use of aptamers in combination with MIPs.

The two described are the only papers which deal with the aptamer-based detection of enrofloxacin, so there exists potential for further research. 

#### 2.5.4. Ofloxacin

Ofloxacin (Figure 6d) is a second-generation fluoroquinolone, used in bacterial infections of the respiratory tract and the gastrointestinal tract [184].

Reinemann et al. [185] searched for aptamer sequences specific for ofloxacin and, furthermore, determined the dissociation constant (K_D_ value) of the aptamer-target system.

In 2017 Pilehvar et al. [184] developed a rapid, stable, and sensitive label-free electrochemical aptasensor for ofloxacin detection based on the immobilization of the specific aptamer on AuNPs. The most important data of the two aptasensors are summarized in Table 13.

There are no more reports about aptasensors for ofloxacin detection.

### 2.6. Lincosamide

**Figure 7:**
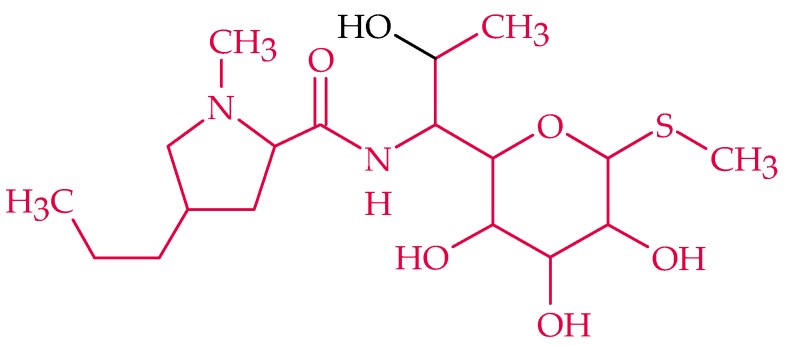
Chemical structure of lincomycin. The basic structure of the anthracyclines is marked in red.

Lincosamides, called acylaminopyranosides due to their chemical structure, bind to the 50S subunit of the bacterial ribosomes and block the enzyme peptidyltransferase, resulting in an interrupted chain elongation during protein biosynthesis [56]. Lincosamides are frequently used in the case of staphylococcal, streptococcal, and pneumococcal infections [57]. Three representatives exist: the natural lincomycin (Figure 7) and two semi-synthetic derivates, clindamycin and pirlimycin [5]. The structure, which is common to all anthracyclines, is marked in red in Figure 7. Anthracyclines are applied especially if a penicillin allergy exists [57].

Lincomycin was the first discovered lincosamide, isolated from *Streptomyces lincolnensis* in a soil sample from Lincoln (Nebraska) [5,57]. It is preferable for the treatment of bone marrow inflammation and wound and respiratory infections [57].

To the best of our knowledge, there is just one report of sensor assay for lincomycin with a dual recognition system comprising a MIP and aptamers [186]. They used the AuNP-functionalized GO nanocomposite for signal amplification, and C-dots, which were modified onto the lincomycin-specific aptamers, serving as a signal indicator and exhibiting enhanced signal intensity in the absence of lincomycin. Electrogenerated chemiluminescence resonance energy transfer was observed between Au-GO and C-dots. After the C-dots accepted the energy, they acted as a signal indicator and exhibited enhanced signal intensity in the presence of the target lincomycin. The results confirmed that the combined characteristics of the specific molecular recognition properties of aptamers and MIPs enhance the recognition ability and cause a high specificity towards their target. The most important data of the discussed aptasensor are given below in Table 14.

### 2.7. Tetracyclines

**Figure 8:**
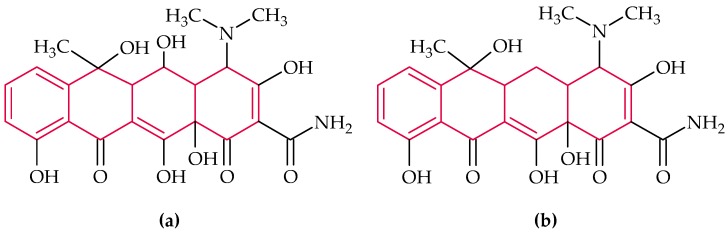
Chemical structure of: (**a**) oxytetracycline and (**b**) tetracycline. The basic structure of the tetracyclines is marked in red.

Tetracyclines are the most widely used antibiotics besides penicillins which are of great economic importance due to their broad-spectrum activity (acting against Gram-positive, Gram-negative bacteria, *Rickettsiae*, *Mycoplasmas*, *Leptospira*, and some large viruses) and their low toxicity [56,57]. In some countries, they are widely used as nutritive antibiotics in poultry and pig fattening, which encourages resistance development. Tetracyclines inhibit protein biosynthesis by binding to the 50S subunit of the ribosomes. They are formed exclusively by *Streptomyces*. Their name derives from their basic structure, which consists of four linearly arranged six-rings (marked in red in Figure 8).

#### 2.7.1. Oxytetracycline

Oxytetracycline (Figure 8a) is the primary product in the formation of tetracyclines by *Streptomyces* [57].

Niazi et al. [187] selected oxytetracycline-specific ssDNA aptamers by Flu-Mag SELEX, which was later used in a variety of studies which deal with oxytetracycline detection [188,189,190,191,192]. In the Flu-Mag SELEX method fluorescent labels for DNA quantification and magnetic beads for target immobilization are used for aptamer selection [193]. In further investigation they selected ssDNA aptamers specific for tetracycline, oxytetracycline, and doxycycline [194].

Kwon et al. [195] truncated 76-mer ssDNA aptamers with high affinity and specificity for oxytetracycline, selected by SELEX, to a unique shortened 8-mer ssDNA, by selection of the nucleotide bases which exhibit high homogeneity in accordance with their conserved regions. By utilization of the shortened aptamer, an ultrasensitive (Table 15) colorimetric oxytetracycline detection based on unmodified AuNPs was possible. The truncated aptamer was used in other studies [196].

An aptamer-based cantilever array sensor for the detection of oxytetracycline at nanomolar concentrations was introduced by Hou et al. [197]. The sensing cantilevers were functionalized with SAMs of the specific aptamers while the reference cantilevers were modified with 6-mercapto-1-hexanol (MCH) SAMs to eliminate the influence of environmental disturbances, such as temperature and non-specific adsorption. 

Meng et al. [196] designed an ultrasensitive surface enhanced Raman scattering (SERS) aptasensor for the detection of oxytetracycline on the basis of the Raman hot spot between gold nanoparticles (AuNPs) (13 nm and 80 nm diameter respectively) linked by a DNA sequence. Advantages of SERS, a molecular fingerprint spectrum, are, amongst others, ultrasensitive and non-invasive probing, compatibility with aqueous solutions, minimal sample preparation, and label-free monitoring of analytes in complex matrices. Thiolated stem-loop DNA, containing the oxytetracycline specific aptamer, was immobilized onto the surface of 80 nm AuNPs and, subsequently, the 13 nm AuNPs were functionalized with the Raman reporter molecule 4-mercaptobenzoic acid. Between the 80 nm AuNPs and the 13 nm AuNPs a SERS hot spot was formed, which is a highly-localized region of intense local field enhancement. In the presence of oxytetracycline, the aptamer preferentially bound to it, leading to a partial dehybridization of the DNA. In consequence, the 13 nm AuNPs approach the 80 nm AuNPs more closely and the Raman intensity increased significantly. 

Two colorimetric aptasensors for the detection of oxytetracycline based on the immobilization of specific aptamers onto AuNP surfaces were reported [190,198].

Based on quenching ability of rGO [34], GO sheets [199], and GO hydrogel [200], several fluorometric assays for oxytetracycline detection are reported, which are described in Table 15.

Consequently, extensive studies have been carried out to improve the performance of aptasensors for detection of oxytetracycline based on luminescence [40,93,201], and photoelectrochemical [202,203] and electrochemical sensing systems [107,173,174,188,204,205,206], which are summarized in Table 15. As seen, the proposed aptasensor based on ultrasensitive surface enhanced Raman scattering reached the lowest LOD for oxytetracycline detection [196], followed by Chen et al. [174] with an amperometric one (Table 15).

#### 2.7.2. Tetracycline

Tetracycline (Figure 8b) is used in veterinary medicine and treatment, as well as the prevention of microbial infections, such as respiratory tract infections, arthritis, and severe acne [207]. In particular, it has been used as a feed additive to promote the growth of livestock in the agriculture sector [208].

In 2008 Niazi et al. [194] identified tetracycline group-specific ssDNA aptamers by modified SELEX (Toggle-SELEX combined with Flu-Mag SELEX) and Müller et al. [209] characterized tetracycline-specific RNA aptamers and their ligand binding properties.

Kwon et al. [195] truncated 76-mer ssDNA aptamers with high affinity and specificity for oxytetracycline to a unique shortened 8-mer ssDNA with selectivity to oxytetracycline, tetracycline, doxycycline, and chlortetracycline. 

Aslipashaki et al. [207] developed an aptamer-based solid-phase extraction followed by electrospray ionization-ion mobility spectrometry (ESI-IMS) for tetracycline separation and detection in biological fluids via covalent binding of aptamers onto CNBr-activated sepharose. 

Jeong and Rhee Paeng [210] introduced a competitive enzyme-linked aptamer assay (ELAA) for the determination of tetracycline residue in bovine milk using two different aptamers individually, one 76mer DNA and a 57mer RNA aptamer. The RNA aptamer featured a higher affinity to tetracycline than the DNA aptamer, and also the LODs obtained for the RNA aptamer were lower than the one for the DNA aptamer, as well as in buffer and in milk (Table 16).

Since tetracycline detection is very important in food safety, plenty of research has been devoted to the development of sensitive, selective, and specific aptasensors on the basis of different sensing methods, like colorimetric [195,211,212,213,214,215], surface-enhanced Raman spectroscopic [216,217], FAM-labelled and label-free [218,219,220,221] fluorometric, photoelectrochemical [208,222,223], electrochemiluminescent [93], impedimetric and amperometric electrochemical [41,224,225,226,227,228,229,230,231,232,233,234,235,236,237,238,239] methods. Among them, the proposed electrochemical aptasensors by Jahanbani’s team [236] showed ultrahigh sensitivity towards tetracycline. They fabricated two similar electrochemical aptasensors based on the immobilization of aptamers on the surface of a modified carbon paste electrode with oleic acid (aptasensor I), and a magnetic bar carbon paste electrode with Fe_3_O_4_ magnetic nanoparticles and oleic acid (aptasensor II). The aptasensors II showed a wider dynamic range and lower LODs (3.8 fM and 0.31 nM with electrochemical impedance spectroscopy (EIS) and differential pulse voltammetry (DPV) methods, respectively) than aptasensor I.

Summarized, around half of all developed aptamer-based biosensors for the detection of tetracycline are based on electrochemical sensor principles (either impedimetric or amperometric). Colorimetric aptasensors are frequently used, mainly due to their simple handling and evaluation. In almost all of the mentioned studies, the same tetracycline-specific ssDNA aptamer was used, which was selected and investigated by Niazi et al. [194]. The summarized data in Table 16 shows, in comparison between the RNA aptamer [210] and the ssDNA aptamers applied for tetracycline detection, that the RNA aptamer featured a lower K_D_ and, thus, a much higher affinity to tetracycline than the DNA aptamers [210]. By shortening the ssDNA sequence to the possible minimum with sufficient affinity, Kwon et al. [195] managed to achieve a similar high affinity of the ssDNA aptamer to the target as Jeong et al. [210] reached with the RNA aptamer. 

### 2.8. Sulfonamides

**Figure 9:**
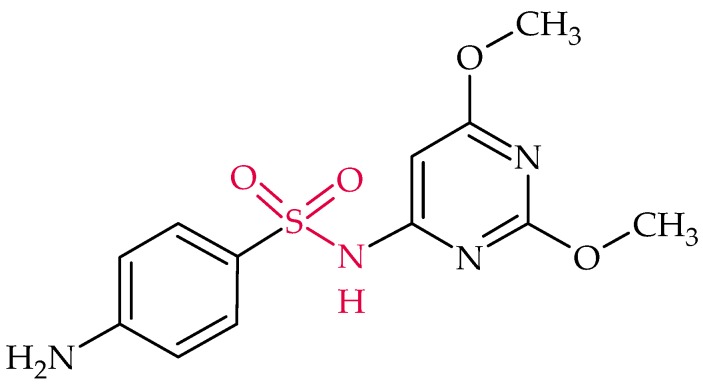
Chemical structure of sulfadimethoxine. The sulfonamide group is marked in red.

Sulfonamides were the first synthetic antibiotics [145]. As the name suggests they are characterized by their sulfonamide group (marked in red in Figure 9). As analogues of *p*-aminobenzoic acid they interfere with the synthesis of folic acid [5]. Sulfonamides act against enterobacteria, like *Escherichia coli* or *Salmonella*, and are mainly used to treat urinary tract infections and pneumonia.

#### Sulfadimethoxine

Sulfadimethoxine (Figure 9) is a cheap broad-spectrum antibiotic that is effective against bacterial and coccidial infections and used for treatment (and prevention) of poultry diseases [243,244].

Song et al. [243] selected aptamers specific for sulfadimethoxine by magnetic bead-based SELEX and identified the one with the highest affinity towards its target. The developed aptamer was utilized in various further work [244,245,246,247,248,249]. According to the data of Table 17, most of the developed aptasensors for sulfadimethoxine detection are colorimetric [75,244,245,246,247]. However, there are some reports on the aptasensors based on fluorometric [248] and photoelectrochemical sensing assays [249]. As seen, the proposed photoelectrochemical aptasensing platform based on graphene-doped Bi_2_S_3_ nanorods, as photoactive materials, compared to other aptamer biosensing assays shows the higher sensitivity towards sulfadimethoxine with a detection limit of 0.55 nM [249].

## 3. Summary

In recent years widespread and uncontrolled usage of antibiotics and, accordingly, their resistance has emerged as a serious problem. Therefore, simple, sensitive, robust, and rapid methods for evaluation of antibiotics and their residues are needed for an on-site screening analysis. The most used conventional methods for antibiotic detection are instrumental ones, such as capillary electrophoresis (CE), gas chromatography (GC), and liquid chromatography (LC), or coupled with mass spectrometry (LC-MS). Despite their wide range of applications, these methods usually have limitations, such as expensive laboratory instruments, require skilled technicians, and require time consuming separation/sample preparation methodologies. Biosensors are considered as ideal alternatives to detect antibiotics in view of their superiority, such as rapid detection, high selectivity, and in situ applications. Therefore, the development of various biosensors and the design of several new signal transduction schemes is a rapidly growing field in biological, clinical, and environmental sciences.

Among different biosensors, the aptamer-based biosensors (aptasensors) are promising tools. Electrochemical aptamer biosensors compared to other developed aptasensors are the most common ones used for antibiotic detection, because of their operational simplicity, high sensitivity, portability, and low cost. Since antibiotics are most often not electrochemically active by themselves, redox tags like methylene blue, ferrocene, or the commonly used Fe^2+^/Fe^3+^ system must be added. Target-induced strand displacement is one of the most widely used signal transduction strategies in aptamer-based biosensors for antibiotic detection. The challenge in replacement reactions is that the affinity of the aptamer towards the target must be stronger than to the complementary DNA (cDNA). The described indirect measuring methods, in which the target is immobilized, are usable for the proof of the function of the developed measurement method, but irrelevant for practical application.

Often a once-established aptamer sequence, specific for a target, is used for almost all further studies. In general, the 5′-end of the aptamer sequence is preferred for immobilization and the 3′-end for labeling (e.g., with FAM) of the aptamer. To evaluate the specificity of the used aptamer towards its target, structurally similar derivates and possible interfering substances are introduced into the sensing system and their influence onto the signal and the detection of the actual target are investigated. In most of the developed aptasensors DNA aptamers were used, and just a few of the mentioned papers dealt with RNA-aptamers. RNA aptamers featured lower K_D_ values and, thus, a higher affinity to their target than DNA aptamers, but RNA is attacked and degraded faster. As shown in the tables, and also compared with the standard HPLC or ELISA methods, sensitivity and selectivity of many aptasensors are acceptable.

The most commonly investigated antibiotics detected by an aptasensor are kanamycin, chloramphenicol, tetracycline, and oxytetracycline. Therefore, a great potential for developing aptamers for other antibiotics with high affinity and specificity exists. Finally, regarding the advantages of aptamers over antibodies, aptamer-based sensors have the potential for clinical/commercial applications and point-of-care detection. 

## Figures and Tables

**Table 1:** Aptamer sequence, dissociation constant (K_D_), limit of detection (LOD), real sample analysis (RSA), and realized sensor type and measuring method for ampicillin, mentioned in the corresponding references (Ref). AC = alternating current, AEC = amperometric electrochemical, apt = aptamer, bsa = bovine serum albumin, cDNA = complementary DNA, CO = colorimetric, CV = cyclic voltammetry, DPV = differential pulse voltammetry, EBFC = enzyme biofuel cell, EIS = electrochemical impedance spectrometry, FAM = fluorescein amidite, FL = fluorometric, hu = human urine, IEC = impedimetric electrochemical, MB = methylene blue, m = milk, OCV = open circuit voltage, rw = river water, sa = salvia, SWV = square wave voltammetry, and w = water.

5′ Linker and Spacer	Aptamer Sequence 5′→3′	3′ Linker and Spacer	K_D_ (nM)	LOD (nM)	RSA	Sensor Type/Method	Ref. ^1^
FAM	I: GCG GGC GGT TGT ATA GCG G II: TTA GTT GGG GTT CAG TTG G III: CAC GGC ATG GTG GGC GTC GTG	-	I: 13.4 II: 9.8 III: 9:4	I: 1.4 (dw, FL) I: 5.7 (m, FL) I: 14.3 (dw, CO) I: 28.6 (m, CO)	m	FL, CO/UV–VIS	[58]
-	apt I: GCG GGC GGT TGT ATA GCG GTT TTT TT apt II: GCG GGC GGT TGT ATA GCG GTT TTT TT cDNA I: AAC CGC CCG CTT TC CTC AGC cDNA II: AAC CGC CCG CTT TAC CTC AGC cDNA III: AAC CGC CCG CTT TAC CTC AGC A cDNA IV: AAC CGC CCG CTT TAC CTC AGC A cDNA V: ACC GCC CGC TTT ACC TCA GCA cDNA VI: CAA CCG CCC GCT TTA CCT CAG CA cDNA VII: ACA ACC GCC CGC TTT ACC TCA GCA	apt I: SH apt II:	-	0.2 (b) (0.07 × 10^−6^ g/L)	rw	FL	[60]
NH_2_-C_6_	GCG GGC GGT TGT ATA GCG G	-	13.4	0.1 (b)	m	IEC/EIS	[58,59]
poly(T)-poly(C)	GCG GGC GGT TGT ATA GCG G	-	-	0.1 (b)	-	IEC/EIS	[61,62]
-	-	-	-	0.001 (b)	m	AEC/DPV	[63]
MB	GCG GGC GGT TGT ATA GCG G	A_10_	-	0.004 (b)	m	AEC/DPV	[64]
apt: SH cDNA: SH	TGG GGG TTG AGG CTA AGC CGA C cDNA: GTC TTA GCC TCA ACC CCC A	-	-	0.00038 (b)	m	AEC/DPV	[65]
SH-(CH_2_)_6_	TTA GTT GGG GTT CAG TTG G	MB		1000 (AC) 30,000 (SWV)	bsa, sa, m	AEC/AC, SWV	[66]
SH-(CH_2_)_6_	apt: TTA GTT GGG GTT CAG TTG G cDNA I: CCA ACT AA cDNA II: CCC AAC TA cDNA III: CCC AAC TAA cDNA IV: CCC CAA CTA cDNA V: CCC CAA CTA A cDNA VI: ACC CCA ACT AA cDNA VII:AAC CCC AAC TAA cDNA VIII: GAA CCC CAA CTA A cDNA IX: TGA ACC CCA ACT AA	MB	-	30 (b)	hu, w, m, sa	AEC/AC	[67]
apt: NH_2_-(CH_2_)_6_ cDNA: SH-(CH_2_)_6_	TTT TGC GGG CGG TTG TAT AGC GG cDNA: TTT TTT TTT CCG CTA TAC AAC CGC C	-	-	0.003 (b)	m	EBFC/CV, OCV	[68]

^1^ When naming several references, the first always describes the realized sensor with associated LOD; aptamer sequence(s) and/or associated K_D_ values are derived from the additional reference(s).

**Table 2:** Aptamer sequence, dissociation constant (K_D_), limit of detection (LOD), real sample analysis (RSA), and realized sensor type and measuring method for penicillin G, mentioned in the corresponding references (Ref). b = buffer, EIS = electrochemical impedance spectrometry, FAM = fluorescein amidite, FL = fluorometric, and IEC = impedimetric electrochemical.

5′ Linker and Spacer	Aptamer Sequence 5′→3′	3′ Linker and Spacer	K_D_ (nM)	LOD (nM)	RSA	Sensor Type/Method	Ref ^1^
-	GGG AGG ACG AAG CGG AAC GAG ATG TAG ATG AGG CTC GAT CCG AAT GCG TGA CGT CTA TCG GAA TAC TCG TTT TTA CGC CTC ATA AGA CAC GCC CGA CA	-	-	0.49 (b) (0.17 × 10^−6^ g/L)	m	IEC/EIS	[70]
FAM	GGG TCT GAG GAG TGC GCG GTG CCA GTG AGT	-	383.4	9.2 (b)	m	FL	[71]
NH_2_	CTG AAT TGG ATC TCT CTT CTT GAG CGA TCT CCA CA	-	-	0.057 (b)	m	IEC/EIS	[69] ²

^1^ When naming several references, the first always describes the realized sensor with associated LOD; aptamer sequence(s) and/or associated K_D_ values are derived from the additional reference(s). ^2^ The exact subcategory of the substance is not mentioned.

**Table 3:** Aptamer sequence, dissociation constant (K_D_), limit of detection (LOD), real sample analysis (RSA), and realized sensor type and measuring method for gentamicin, mentioned in the corresponding references (Ref). AEC = amperometric electrochemical, hs = human serum, MB = methylene blue, and SWV = square wave voltammetry.

5′ Linker and Spacer	Aptamer Sequence 5′→3′	3′ Linker and Spacer	K_D_ (nM)	LOD (nM)	RSA	Sensor Type/Method	Ref ^1^
I: SH II: SH III: SH	I: GGG ACU UGG UUU AGG UAA UGA GUC CC II: (fully O-methylated) GGG ACU UGG UUU AGG UAA UGA GUC CC III: GGG ACT TGG TTT AGG TAA TGA GTC CC	I: NH-MB II: NH-MB III: NH-MB	I: 72,000 II: ≈ 80,000 III: ≈ 200,000	-	hs	AEC/SWV	[72,73]

^1^ When naming several references, the first always describes the realized sensor with associated LOD; aptamer sequence(s) and/or associated K_D_ values are derived from the additional reference(s).

**Table 4:** Aptamer sequence, dissociation constant (K_D_), limit of detection (LOD), real sample analysis (RSA), and realized sensor type and measuring method for kanamycin, mentioned in the corresponding references (Ref). apt = aptamer, AEC = amperometric electrochemical, b = buffer, bs = blood serum, c = chicken, CA = chronoamperometry, CAN = cantilever, cap = capture probe, cDNA = complementary DNA, CO = colorimetric, cy = cyanine dye, DPV = differential pulse voltammetry, ECL = electrochemiluminescent, EIS = electrochemical impedance spectrometry, f = fish, FAM = fluorescein amidite, FL = fluorometric, hs = human serum, IEC = impedimetric electrochemical, LCA = liquid crystal assay, lw = lake water, m = milk, MB = methylene blue, p = pork, PEC = photoelectrochemical, rs = rat serum, ROX = 6-carboxyl-x-rhodamine, SWV = square wave voltammetry, and ww = waste water.

5′ Linker and Spacer	Aptamer Sequence 5′→3′	3′ Linker and Spacer	K_D_ (nM)	LOD (nM)	RSA	Sensor Type/Method	Ref ^1^
SH-(CH_2_)_6_	TGG GGG TTG AGG CTA AGC CGA C	-	-	50000 (b)	-	CAN	[94]
-	TGG GGG TTG AGG CTA AGC CGA	-	78.8	25 (b)	-	CO/UV–VIS	[74]
-	TGG GGG TTG AGG CTA AGC CGA	-	78.8	-	-	CO/UV–VIS	[74,75]
-	TGG GGG TTG AGG CTA AGC CGA	-	8.38	1.49 (b)	-	CO	[32,74]
SH-(CH_2_)_6_	TGG GGG TTG AGG CTA AGC CGA	-	-	0.014 (b)	m	CO/UV–VIS	[74,95]
-	TGG GGG TTG AGG CTA AGC CGA	-	-	4.5 (b) (2.6 × 10^−6^ g/L)	m	CO	[77]
-	CGG AAG CGC GCC ACC CCA TCG GCG GGG GCG AAG CTT GCG	-	-	3.35 (b)	m	CO	[78,85]
apt: SH-(CH_2_)_6_ cDNA I: SH-(CH_2_)_6_ cap: biotin	apt: TGG GGG TTG AGG CTA AGC CGA cDNA I: TCA GTC GGC TTA GCC GTC CAA CGT CAG ATC C cap: CCG ATG GAT CTG ACG T	apt: biotin		0.0778 (b)	m, h	CO	[79]
-	TGG GGG TTG AGG CTA AGC CGA	NH_2_-(CH_2_)_6_	-	<1 (b)	-	LCA	[74,80]
apt: biotin cDNA: FAM	apt: AGA TGG GGG TTG AGG CTA AGC CGA cDNA: CTT AGC CTC AAC CCC CAT CT	-	-	0.612 (b) 0.453 (rs)	rs	FL	[81]
apt: biotin cDNA: ROX	apt: TGG GGG TTG AGG CTA AGC CGA cDNA: TCG GCT TAG CCT CAA CCC CCA	-	-	1.58 (b) (0.92 × 10^−6^ g/L)	m, h, p	FL	[74,82]
-	AGA TGG GGG TTG AGG CTA AGC CGA	-	-	0.321 (b) 0.476 (m) 0.568 (rs)	m, rs	FL	[58,83,90]
-	apt: TGG GGG TTG AGG CTA AGC CGA	-	-	59 (b)	m	FL	[74,89]
NH_2_	AGA TGG GGG TTG AGG CTA AGC CGA	-	-	0.009 (b) 0.018 (bs)	bs	FL	[84]
II: FAM	I: ATG CGG ATC CCG CGC GAC CAA CGG AAG CGC GCC ACC CCA TCG GCG GGC GCG AAG CTT GCG C II: CGG AAG CGC GCC ACC CCA TCG GCG GGC GCG AAG CTT GCG	-	II: 92.3	I: 6.25 (b) II: 6.25 (b) II: 0.001 (st) II: 0.1 (bs) II: 0.02 (m)	m, bs	FL	[85]
apt I: FAM	apt I: TGG GGG TTG AGG CTA AGC CGA apt II: TGG GGG TT FAM GAG GCT AAG CCG A apt III: TGG GGG TTG AGG CTA AGC CGA cDNA I: AAC CCC cDNA II: AAC CCC A cDNA III: AAC CCC CAA CT	cDNA I: FAM cDNA II: FAM cDNA III: FAM	-	0.4 (b)	m	FL	[86]
NH_2_-C_6_	TGG GGG TTG AGG CTA AGC CGA C	-	-	1100 (b)	m	FL	[87]
apt II: Cy3 apt III: Cy5 anchor apt: NH_2_ cDNA II: Cy3	apt I: TGG GGG TTG AGG CTA AGC CGA apt II: TGG GGG TTG AGG CTA AGC CGA apt III: TGG GGG TTG AGG CTA AGC CGA apt IV: TGG GGG TTG AGG CTA AGC CGA anchor apt: TTT TTT TGG GGG TTG AGG CTA AGC CGA cDNA I: TAG CCT CAA cDNA II: TCG GCT TAG CCT	apt IV: Cy3 cDNA I: Cy3		26 (b)	m	FL	[74,90]
-	TGG GGG TTG AGG CTA AGC CGA	-	78.8	143 (b)	f	ECL	[74,91]
SH-(CH_2_)_6_-T_5_	TGG GGG TTG AGG CTA AGC CGA G-quadruplex: GGT TGG TGT GGT TGG TAG CCT CAA GGT TGG TGT GGT TGG	-	-	0.045 (b)	m	ECL	[92]
apt: biotin cDNA: SH-(CH_2_)_6_	apt: TGG GGG TTG AGG CTA AGC CGA cDNA: TTA GCC TCA A	-	-	0.034 (b) (0.002 × 10^−6^ g/L)	m	ECL	[93]
-	TGG GGG TTG AGG CTA AGC CGA	-	-	0.2 (b)	-	PEC	[96]
SH-(CH_2_)_6_	TGG GGG TTG AGG CTA AGC CGA	-	-	0.1 (b)	-	PEC/EIS, CA	[97]
SH-(CH_2_)_6_	TGG GGG TTG AGG CTA AGC CGA	-	-	7.2 (b) (3.5 × 10^−6^ g/L)	m	PEC/EIS	[97,98]
-	TGG GGG TTG AGG CTA AGC CGA	-	-	1.0 (b)	m	IEC/EIS	[76]
-	TGG GGG TTG AGG CTA AGC CGA	-	-	0.23 (0.11 × 10^−6^ g/L)	m	IEC/EIS	[74,99]
I: SH II: SH III: SH	I: GGG ACU UGG UUU AGG UAA UGA GUC CC II: (fully O-methylated) GGG ACU UGG UUU AGG UAA UGA GUC CC III: GGG ACT TGG TTT AGG TAA TGA GTC CC	I: NH-MB II: NH-MB III: NH-MB	I: 281,000 II: ≈ 450,000 III: ≈ 600,000	-	hs	AEC/SWV	[72,73]
NH_2_	TGG GGG TTG AGG CTA AGC CGA C	-	78.8	9.4 ± 0.4 (b) 10.8 ± 0.6 (m)	m	AEC/SWV	[100]
I: NH_2_ II: biotin	I: TGG GGG TTG AGG CTA AGC CGA C II: TGG GGG TTG AGG CTA AGC CGA C	-	-	5.8 (b)	m	AEC/DPV	[101]
I NH_2_ II biotin	I: TGG GGG TTG AGG CTA AGC CGA C II: TGG GGG TTG AGG CTA AGC CGA C	-	-	8.6 (b)	m	AEC/DPV	[102]
biotin	TGG GGG TTG AGG CTA AGC CG	-	-	7.9 (b) (4,6 × 10^−6^ g/L)	m	AEC/DPV	[103]
NH_2_	AGA TGG GGG TTG AGG CTA AGC CGA	-	-	0.0037 (b)	m	AEC/DPV	[104]
PO_4_	AGA TGG GGG TTG AGG CTA AGC CGA	-		0.87 (b)	m, p, c	AEC/DPV	[105]
NH_2_	AGA TGG GGG TTG AGG CTA AGC CGA	-	-	0.00042 (b)	m, p, c	AEC/DPV	[106]
-	TCT GGG GGT TGA GGC TAA GCC GAC	(CH_2_)_6_-NH_2_	78.8	0.00015 (b)	m	AEC/SWV	[101,107]
apt: SH cDNA: apt	apt: TGG GGG TTG AGG CTA AGC CGA C cDNA: GTC GGC TTA CGG TCA ACC CCC A	-	-	0.01 (b) (0.005 × 10^−6^ g/L)	m	AEC/SWV	[108]
-	TGG GGG TTG AGG CTA AGC CG	-	-	0.00074 (b)	m	AEC/DPV	[109]
-	TGG GGG TTG AGG CTA AGC CGA C	-	-	0.0000013 (b)	m	AEC/DPV	[110]
apt: NH_2_-(CH_2_)_6_ cDNA: NH_2_-(CH_2_)_6_	apt: TGG GGG TTG AGG CTA AGC CGA C cDNA: CGT TAG CCT CAA CCC	-	-	0.00016 (b)	m	AEC/SWV	[49]
SH	TGG GGG TTG AGG CTA AGC CGA	-	-	0.00137 (b) (0.008 × 10^−9^ g/L)	m	AEC/DPV	[111]
apt I: SH	apt: TGG GGG TTG AGG CTA AGC CGA			0.000035 (b)	m	AEC/SWV	[74,112]
FAM	ATA CCA GCT TAT TCA ATT AGC CCG GTA TTG AGG TCG ATC TCT TAT CCT ATG GCT TGT CCC CCA TGG CTC GGT TAT ATC CAG ATA GTA AGT GCA ATC T	-	3900	5000 (ww)	ww	FL	[113] ^2^
FAM	TGG GGG TTG AGG CTA AGC CGA	-	115 ± 2.76	0.3 (b)	m	FL	[74,114] ^2^
-	apt: TGG GGG TTG AGG CTA AGC CGA mut I: TGG AGG TTG AG CTA AGC CGA mut II: TGG AGG TTG AGG CTA AGC CGA mut III: TGG AGG TTG AAG CTA AAC CGA mut IV: TAA AAA TTA AAA CTA AAC CAA	-	-	0.3 (b)	m	FL	[74,115] ^2^
NH_2_-C_6_	TGG GGG TTG AGG CTA AGC CGA	-	78.8	10 (b)	m	IEC/EIS	[58,59] ^2^
cDNA I: ferrocene-(CH_2_)_6_ cDNA II: SH-(CH_2_)_6_	apt I: TGG GGG TTG AGG CTA AGC CGA GTC ACT AT cDNA I: GTG ACT CGG CTT apt II: TGG GGG TTG AGG CTA AGC CGA GTC ACT AT cDNA II: TAT GTG ACT CGG CTT	apt I: (CH_2_)_3_-SH apt II: (CH_2_)_3_-ferrocene	78.8	1.0 (b)	lw	IEC/EIS	[74,116] ^2^

^1^ When naming several references, the first always describes the realized sensor with associated LOD; aptamer sequence(s) and/or associated K_D_ values are derived from the additional reference(s). ^2^ Kanamycin A was investigated.

**Table 5:** Aptamer sequence, dissociation constant (K_D_), limit of detection (LOD), real sample analysis (RSA), and realized sensor type and measuring method for neomycin B, mentioned in the corresponding references (Ref). b = buffer, FAM = fluorescein amidite, FIS = Faradaic impedance spectroscopy, FL = fluorometric, IEC = impedimetric electrochemical, m = milk, and SPR = surface plasmon resonance spectroscopy.

5′ Linker and Spacer	Aptamer Sequence 5′→3′	3′ Linker and Spacer	K_D_ (nM)	LOD (nM)	RSA	Sensor Type/Method	Ref ^1^
FAM	GGA CUG GGC GAG AAG UUU AGU CC	(T)_15_–(A)_12_	115 ± 25	10 (m)	m	FL	[118,119]
-	(fully O-methylated) GGC CUG GGC GAG AAG UUU AGG CC	-	-	<1000 (b)	m	IEC/FIS	[120]
-	(fully O-methylated) GGC CUG GGC GAG AAG UUU AGG CC	-	2500 ± 900	5 (b, SPR)	-	IEC/FIS SPR	[121]

^1^ When naming several references, the first always describes the realized sensor with associated LOD; aptamer sequence(s) and/or associated K_D_ values are derived from the additional reference(s).

**Table 6:** Aptamer sequence, dissociation constant (K_D_), limit of detection (LOD), real sample analysis (RSA), and realized sensor type and measuring method for tobramycin, mentioned in the corresponding references (Ref). AEC = amperometric electrochemical, b = buffer, bs = blood serum, bsa = bovine serum, albumin, CA = chronoamperometry, ce = chicken egg, CO = colorimetric, DPV = differential pulse voltammetry, FIS = Faradaic impedance spectroscopy, h = honey, hs = human serum, IEC = impedimetric electrochemical, m = milk, MB = methylene blue, SPR = surface plasmon resonance, and SWV = square wave voltammetry.

5′ Linker and Spacer	Aptamer Sequence 5′→3′	3′ Linker and Spacer	K_D_ (nM)	LOD (nM)	RSA	Sensor Type/Method	Ref ^1^
SH	TCC GTG TAT AGG TCG GGT CTC TTG CCA ACT GAT TCG TTG AAA AGT ATA GCC CCG CAG GG	-	260	500 (b) 3400 (bs)	bs	SPR	[122,123]
-	I: TAG GGA ATT CGT CGA CGG ATC CAT GGC ACG TTA TGC GGA GGC GGT ATG ATA GCG CTA CTG CAG GTC GAC GCA TGC GCC G II: CGT CGA CGG ATC CAT GGC ACG TTA TGC GGT ATG ATA GCG CAG GTC GAC G III: CGT CGA CGG ATC CAT GGC ACG TTA TAG GTC GAC G	-	I: 56.9 II: 46.8 III: 48.4	37.9 (b)	h	CO	[124]
-	GGG ACT TGG TTT AGG TAA TGA GTC CC	-	-	23.3 (b)	m, ce	CO	[125]
-	I: (O-methylated RNA except **U12** position) GGC ACG AGG UU**U** AGC UAC ACU CGU GCC II: (fully O-methylated) GGC ACG AGG UUU AGC UAC ACU CGU GCC	-	I: 600 II: 400	I: 700 (b) II: 400 (b)	hs	IEC/FIS	[73,126]
I: SH II: SH III: SH	I: GGG ACU UGG UUU AGG UAA UGA GUC CC II: (fully O-methylated) GGG ACU UGG UUU AGG UAA UGA GUC CC III: GGG ACT TGG TTT AGG TAA TGA GTC CC	I: NH-MB II: NH-MB III: NH-MB	I: 319,000 II: ≈ 180,000 III: ≈ 1,380,000	-	hs	AEC/SWV	[72,73]
biotin	(O-methylated except **U12** position) GGC ACG AGG UU**U** AGC UAC ACU CGU GCC	-	-	5000 (b)	-	AEC/DPV	[73,127]
fluorescein	(O-methylated except **U12** position) GGC ACG AGG UU**U** AGC UAC ACU CGU GCC	-	-	100 (b)	hs	AEC/DPV, CA	[129]
I: SH-C_6_ II: SH-C_6_ III: SH-C_6_ IV: SH-C_6_	I: GGG ACU UGG UUU AGG UAA UGA GUC CC II: ACU UGG UUU AGG UAA UGA GU III: CUU GGU UUA GGU AAU GAG IV: GGG ACU UGG UUU AGG UAA UGA GU	I: MB II: MB III: MB IV: MB	I: 16,000 ± 3000 II: 220 ± 50 III: 510 ± 70 IV: 2900 ± 900 III: 148,000 ± 4000 (s)	-	bsa	AEC/SWV	[73,128]

^1^ When naming several references, the first always describes the realized sensor with associated LOD; aptamer sequence(s) and/or associated K_D_ values are derived from the additional reference(s).

**Table 7:** Aptamer sequence, dissociation constant (K_D_), limit of detection (LOD), real sample analysis (RSA), and realized sensor type and measuring method for streptomycin, mentioned in the corresponding references (Ref). AEC = amperometric electrochemical, b = buffer, bs = blood serum, cap = capture probe, cDNA = complementary DNA, CO = colorimetric, DPV = differential pulse voltammetry, FAM = fluorescein amidite, FL = fluorometric, h = honey, IEC = impedimetric electrochemical, m = milk, PEC = photoelectrochemical, rs = rat serum, and SWV = square wave voltammetry.

5′ Linker and Spacer	Aptamer Sequence 5′→3′	3′ Linker and Spacer	K_D_ (nM)	LOD (nM)	RSA	Sensor Type/Method	Ref ^1^
-	I: GGG GTC TGG TGT TCT GCT TTG TTC TGT CGG GTC GT II: TGA AGG GTC GAC TCT AGA GGC AGG TGT TCC TCA GG III: AGC TTG GGT GGG GCC ACG TAG AGG TAT AGC TTG TT IV: TGT GTG TTC GGT GCT GTC GGG TTG TTT CTT GGT TT	-	I: 199.1 II: 221.3 III: 272.0 IV: 340.6	I: 200 (b) I: 200 (h)	h	CO/UV–VIS	[130]
I: FAM II: FAM III: FAM	I: CCC GTT TAA AGT AGT TGA GAG TAT TCC GTT TCT TTG TGT C II: GTG CGT TAT AAA CTA GTT TTG ATT CAA TGT TGG GTG TGG G III: GGG CCT GTT TTG CCT TCA CGT TCT CTT CCT TGC CGT TCT G	I: biotin II: biotin III: biotin	I: 6.07 II: 8.56 III: 13.14	25 (b)	m, h	CO	[131]
SH-(CH_2_)_6_	TAG GGA ATT CGT CGACGG ATC CGG GGT CTG GTG TTC TGC TTT GTT CTG TCG GGT CGTCTG CAG GTC GAC GCA TGC GCC G	-	-	0.0017 (b) (1∙10^−9^ g/L)	m	CO	[130,132]
SH	TAG GGA ATT CGT CGA CGA ATC CGG GGT CTG GTG TTC TGC TTT GTT CGTB TCG GGT CGT CTG CAG GTC GAC GCA TGC GCC G	-	199.1	86 (b)	m	CO	[130,133]
cDNA: FAM	apt: TAG GGA ATT CGT CGA CGG ATG CGG GGT CTG GTG TTG TGC TTT GTT CTG TCG GGT CGT CTG CAG GTC GAC GCA TGC GCC G cDNA: CGG CGC ATG CGT CGA CCT GCA GAC GAC CCG ACA GAA CAA AGC AGA ACA CCA GAC CCC GGA TCC GTC GAC GAA TTC CCT A	-	-	73.1 (b, CO) 102.4 (bs, CO) 108.7 (m, CO) 47.6 (b, FL) 58.2 (bs, FL) 56.2 (m, FL)	m, bs	CO, FL/UV–VIS	[134]
-	apt: TAG GGA ATT CGT CGA CGG ATG CGG GGT CTG GTG TTG TGC TTT GTT CTG TCG GGT CGT CTG CAG GTC GAC GCA TGC GCC G cDNA: CGG CGCA TGC GTC GAC CTG CAG ACG ACC CGA CAG AAC AAA GCA GAA CAC CAG ACC CCG GAT CCG TCG ACG AAT TCC CTA	-	-	54.5 (b) 71.0 (rs) 76.05 (m)	m, bs	FL	[135]
-	GGG GTC TGG TGT TCT GCT TTG TTC TGT CGG GTC GT	-	-	0.05 (b)	m	FL	[130,141]
-	apt: TAG GGA ATT CGT CGA CGG ATC CGG GGT CTG GTG TTC TGC TTT GTT CTG TCG GGT CGT CTG CAG GTC GAC GCA TGC GCC G cDNA I: CGG CGGC ATG CGT CGA CCT GCA GAC GAC CCG ACA GAA CAA AGC AGA ACA CCA GAC CCC GGA TCC GTC GAC GAA TTC CCT A cDNA II: CAG ACG ACC CGA CAG AAC AAA GCA GAA CAC CAG ACC CCG GAT CCG TCG ACG AAT TCC CTA cDNA III: GAC AGA ACA AAG CAG AAC ACC AGA CCC CGG ATC CGT CGA CGA ATT CCC TA cDNA IV: AGC AGA ACA CCA GAC CCC GGA TCC GTC GAC GAA TTC CCT A	-	-	94 (b)	m, c	FL	[132,142]
-	TAG GGA ATT CGT CGA CGG ATC CGG GGT CTG GTG TTC TGC TTT GTT CTG TCG GGT CGT CTG CAG GTC GAC GCA TGC GCC G	NH_2_	-	0.033 (b)	h	PEC	[136]
-	TAG GGA ATT CGT CGA CGG ATG CGG GGT CTG GTG TTG TGC TTT GTT CTG TCG GGT CGT CTG CAG GTC GAC GCA TGC GCC G	SH	-	0.057∙10^−3^ (b) (0.033∙10^−9^ g/L)	hs	IEC	[137]
-	TAG GGA ATT CGT CGA CGG ATG CGG GGT CTG GTG TTG TGC TTT GTT CTG TCG GGT CGT CTG CAG GTC GAC GCA TGC GCC G	SH	-	11.4 (b) 14.1 (m) 15.3 (rs)	m, rs	AEC/DPV	[143]
cap: SH-(CH_2_)_6_	apt: TAG GGA ATT CGT CGA CGG ATG CGG GGT CTG GTG TTG TGC TTT GTT CTG TCG GGT CGT CTG CAG GTC GAC GCA TGC GCC G cap: GGT GTT GGT GTT cDNA I: GAC AGA ACA AAG CAG AAC ACC A cDNA II: TTC TGT CTC TCG	cDNA II: biotin	-	10 (b)	m	AEC/SWV	[41]
-	TAG GGA ATT CGT CGA CGG ATG CGG GGT CTG GTG TTG TGC TTT GTT CTG TCG GGT CGT CTG CAG GTC GAC GCA TGC GCC G	SH	-	0.036 (b)	m, h	AEC/DPV	[138]
NH_2_	TAG GGA ATT CGT CGA CGG ATG CGG GGT CTG GTG TTG TGC TTT GTT CTG TCG GGT CGT CTG CAG GTC GAC GCA TGC GCC G	-		0.0078 (b)	m	AEC/DPV	[139]
-	TAG GGA ATT CGT CGA CGG ATG CGG GGT CTG GTG TTG TGC TTT GTT CTG TCG GGT CGT CTG CAG GTC GAC GCA TGC GCC G	SH	-	0.028 (b)	m	AEC/DPV	[140]

^1^ When naming several references, the first always describes the realized sensor with associated LOD; aptamer sequence(s) and/or associated K_D_ values are derived from the additional reference(s).

**Table 8:** Aptamer sequence, dissociation constant (K_D_), limit of detection (LOD), real sample analysis (RSA), and realized sensor type and measuring method daunomycin in the corresponding references (Ref). AEC = amperometric electrochemical, b = buffer, CO = colorimetric, DPV = differential pulse voltammetry, ELAA = enzyme-linked aptamer assay, FL = fluorometric, hu = human urine, and SPR = surface plasmon resonance.

5′ Linker and Spacer	Aptamer Sequence 5′→3′	3′ Linker and Spacer	K_D_ (nM)	LOD (nM)	RSA	Sensor Type/Method	Ref ^1^
-	GGG AAT TCG AGC TCG GTA CCA TCT GTG TAA GGG GTA AGG GGT GGG GGT GGG TAC GTC TAG CTG CAG GCA TGC AAG CTT GG	-	20	15 (b) (8.4 × 10^−6^ g/L)	-	FL ELAA SPR	[147]
-	GGG AAT TCG AGC TCG GTA CCA TCT GTG TAA GGG GTA AGG GGT GGG GGT GGG TAC GTC TAG CTG CAG GCA TGC AAG CTT GG	-	20	17.6 (b)	-	CO, FL	[146,147]
poly-TTBA-NH_2_	GGG AAT TCG AGC TCG GTA CCA TCT GTG TAA GGG GTA AGG GGT GGG GGT GGG TAC GTC TAG CTG CAG GCA TGC AAG CTT GG	-	20	0.052 ± 0.002 (b)	hu	AEC/DPV	[147,148]

^1^ When naming several references, the first always describes the realized sensor with associated LOD; aptamer sequence(s) and/or associated K_D_ values are derived from the additional reference(s).

**Table 9:** Aptamer sequence, dissociation constant (K_D_), limit of detection (LOD), real sample analysis (RSA), and realized sensor type and measuring method for chloramphenicol, mentioned in the corresponding references (Ref). AEC = amperometric electrochemical, apt = aptamer, b = buffer, cap = capture probe, cDNA = complementary DNA, CO = colorimetric, d = drugs, DPV = differential pulse voltammetry, ECL = electrochemiluminescent, EIS = electrochemical impedance spectrometry, f = fish, FL = fluorometric, h = honey, hs = human serum, IEC = impedimetric electrochemical, LSV = linear sweep voltammetry, m = milk, PEC = photoelectrochemical, p = pork, rs = rat serum, SPR = surface plasmon resonance, u = urine, and w = water.

5′ Linker and Spacer	Aptamer Sequence 5′→3′	3′ Linker and Spacer	K_D_ (nM)	LOD (nM)	RSA	Sensor Type/Method	Ref ^1^
-	I: ACT TCA GTG AGT TGT CCC ACG GTC GGC GAG TCG GTG GTA G II: ACT GAG GGC ACG GAC AGG AGG GGG AGA GAT GGC GTG AGG T	-	I: 766 II: 1160	-	-	FL	[150]
apt: SH-(CH_2_)_6_ cDNA: SH-(CH_2_)_6_	apt: ACT TCA GTG AGT TGT CCC ACG GTC GGC GAG TCG GTG GTA G cDNA: TTT TCT ACC ACC GAC TCG C	-	766	0.062 (b) (0.02 × 10^−6^ g/L)	f, p	CO/UV–VIS	[150,151]
apt: (CH_2_)_6_ cDNA: SH-(CH_2_)_6_	apt: ACT TCA GTG AGT TGT CCC ACG GTC GGC GAG TCG GTG GTA G cDNA: CTA CCA CCG ACT CGC CGA CCG TGG GAC AAC TCA CTG AAG T	-	-	0.046 (b) (0.015 × 10^−6^ g/L)	m	CO/UV–VIS	[150,152]
apt: (CH_2_)_6_ cDNA: SH-(CH_2_)_6_	apt: ACT TCA GTG AGT TGT CCC ACG GTC GGC GAG TCG GTG GTA G cDNA: CTA CCA CCG ACT CGCG CGA CCG TGG GAC AAC TCA CTG AAG T	-	-	0.00093 (b) (0.3 × 10^−9^ g/L)	m	CO/UV–VIS	[153]
-	ACT TCA GTG AGT TGT CCC ACG GTC GGC GAG TCG GTG GTA G	biotin	-	0.451 (b) 0.697 (m) 0.601 (rs)	m, rs	CO/UV–VIS	[176]
NH_2_-C_6_	AGC AGC ACA GAG GTC AGA TGC ACT CGG ACC CCA TTC TCC TTC CAT CCC TCA TCC GTC CAC CCT ATG CGT GCT ACC GTG AA	-	-	0.098 (b) 0.761 (m)	m	FL	[160]
apt: biotin cDNA: NH_2_	apt: AGC AGC ACA GAG GTC AGA TGA CTT CAG TGA GTT GTC CCA CGG TCG GCG AGT CGG TGG TAG CCT ATG CGT GCT ACC GTG AA cDNA: CGA CCG TGG GAC AAC TCA	-	-	0.031 (b) (0.01 × 10^−6^ g/L)	m	FL	[157]
apt: (CH_2_)_6_ cDNA: SH-(CH_2_)_6_	apt: ACT TCA GTG AGT TGT CCC ACG GTC GGC GAG TCG GTG GTA G cDNA: CTA CCA CCG ACT CGC CGA CCG TGG GAC AAC TCA CTG AAG T	-	-	0.0006 (b) (0.0002 × 10^−6^ g/L)	f	FL	[42,150]
apt: (CH_2_)_6_ cDNA: SH-(CH_2_)_6_ G-quadruplex: SH-(CH_2_)_6_	apt: ACT TCA GTG AGT TGT CCC ACG GTC GGC GAG TCG GTG GTA G cDNA: CTA CCA CCG ACT CGC CGA CCG TGG GAC AAC TCA CTG AAG T G-quadruplex: GGG TAG GGC GGG AA	-	-	0.0015 (b) (0.0005 × 10^−6^ g/L)	m	FL	[150,156]
(CH_2_)_6_	ACT TCA GTG AGT TGT CCC ACG GTC GGC GAG TCG GTG GTA G	-	-	0.001 (b)	f	FL	[150,155]
apt: SH-(CH_2_)_6_	apt: ACT TCA GTG AGT TGT CCC ACG GTC GGC GAG TCG GTG GTA G cDNA: CTA CCA CCG ACT CGC	-	-	0.0003 (b)	m	FL	[43,150]
-	apt: CAA TAA GCG ATG CGC CCT CGC CTG GGG GCC TAG TCC TCT CCT ATG CGT GCT ACC GTG AA cDNAI: TCG CTT ATT GAA AAA AAA AA cDNAII: CAT CGC TTA TTG AAA AAA AAA A cDNAIII: CGC ATC GCT TAT TGA AAA AAA AAA	cDNAI: biotin cDNAII: biotin cDNAIII: biotin	32.24	0.31 (b)	m	FL	[158]
SH-(CH_2_)_6_	ACT TCA GTG AGT TGT CCC ACG GTC GGC GAG TCG GTG GTA G	-	766	0.093 (b) (0.003 × 10^−6^ g/L)	m	FL	[150,159]
cDNA: SH-(CH_2_)_6_	apt: ACT TCA GTG AGT TGT CCC ACG GTC GGC GAG TCG GTG GTA G cDNA: TTT TTC TAC CAC CGA CTC	apt: COOH	-	0.07 (b)	-	ECL	[161,167]
apt: biotin cDNA: SH-(CH_2_)_6_	apt: TTT TTA GCA GCA CAG AGG TCA GAT GAC TTC AGT GAG TTG TCC CAC GGT CGG CGA GTC GGT AGC CTA TGC GTG CTA CCG TGA A cDNA: CAC GCA TAG GCT ACC A	-	-	0.031 (b) (0.01 × 10^−6^ g/L) 3.094 (m) (1.0 × 10^−6^ g/L)	m	ECL	[149,150]
cDNA: SH-(CH_2_)_6_	apt: ACT TCA GTG AGT TGT CCC ACG GTC GGC GAG TCG GTG GTA G cDNA: CTA CCA CCG ACT C	apt: (CH_2_)_6_-NH_2_	-	0.03 (b)	f	ECL	[162,167]
apt: (CH_2_)_6_	apt: ACT TCA GTG AGT TGT CCC ACG GTC GGC GAG TCG GTG GTA G cDNA: CTC GCC GAC CGT GGG ACA ACT CAC TGA AGT	-	-	0.000034 (b)	f	ECL/SPR	[150,154]
-	ACT TCA GTG AGT TGT CCC ACG GTC GGC GAG TCG GTG GTA G	-	-	3.1 (b)	d	PEC	[177]
NH_2_	ACT TCA GTG AGT TGT CCC ACG GTC GGC GAG TCG GTG GTA G	-	-	0.00036 (b)	m	PEC	[163]
SH-(CH_2_)_6_	AGC AGC ACA GAG GTC AGA TGA CTG AGG GCA CGG ACA GGA GGG GGA GAG ATG GCG TGA GGT CCT ATG CGT GCT ACC GTG AA	-	-	1.76 (b)	-	IEC/EIS	[150,178]
SH-(CH_2_)_6_	AGC AGC ACA GAG GTC AGA TGA CTG AGG GCA CGG ACA GGA GGG GGA GAG ATG GCG TGA GGT CCT ATG CGT GCT ACC GTG AA	-	-	1000 (b)	-	AEC/SWV	[150,164]
SH-(CH_2_)_6_	AGC AGC ACA GAG GTC AGA TGA CTG AGG GCA CGG ACA GGA GGG GGA GAG ATG GCG TGA GGT CCT ATG CGT GCT ACC GTG AA	-	766	1.6 (b) 1.6 (m)	w, m	AEC/SWV	[150,165]
apt: SH-(CH_2_)_6_ cDNA: biotin	apt: TTT TTA GCA GCA CAG AGG TCA GAT GAC TTC AGT GAG TTG TCC CAC GGT CGG CGA GTC GGT GGT AGC CTA TGC GTG CTA CCG TGA A cDNA: TTT TCT ACC ACC GAC TCG C	-	-	0.29 (b)	h	AEC/DPV	[150,166]
NH_2_	ACT TCA GTG AGT TGT CCC ACG GTC GGC GAG TCG GTG GTA G	-	-	0.02 (b)	u, d	AEC/SWV	[150,167]
SH-(CH_2_)_6_	AGC AGC ACA GAG GTC AGA TGA CTT CAG TGA GTT GTC CCA CGG TCG GCG AGT CGG TGG TAG CCT ATG CGT GCT ACC GTG AA	-	-	4.0 (b)	hs	AEC/SWV	[168]
SH-(CH_2_)_6_	AGC AGC ACA GAG GTC AGA TGA CTG AGG GCA CGG ACA GGA GGG CAT GGA GAG ATG GCG	-	766	0.183 (b)	m	AEC/DPV	[150,169]
NH_2_	ACT TCA GTG AGT TGT CCC ACG GTC GGC GAG TCG GTG GTA G	-	-	0.000011 (b) 0.000014 (u)	d	AEC/SWV	[167,170]
apt: SH-(CH_2_)_6_ cDNA: NH_2_-(CH_2_)_6_	apt: ACT TCA GTG AGT TGT CCC ACG GTC GGC GAG TCG GTG GTA G cDNA: ACC ACC GAC TCG CCG	-		0.0009 (b) (0.3 × 10^−9^ g/L)	f	AEC/SWV	[171]
SH-(CH_2_)_6_	AGC AGC ACA GAG GTC AGA TGA CTT CAG TGA GTT GTC CCA CGG TCG GCG AGT CGG TGG TAG CCT ATG CGT GCT ACC GTG AA	-	-	4.0 (b)	hs	AEC/DPV	[172]
cDNA I: SH-(CH_2_)_6_ cDNA II: NH_2_-(CH_2_)_6_	apt: ACT TCA GTG AGT TGT CCC ACG GTC GGC GAG TCG GTG GTA cDNA I: ACA CAA GGG GGC CAC CAC AA cDNA II: TTG TGG TGG CCC CCT TGT GT	cDNA I: (CH_2_)_6_	-	0.46 (b) (0.15 × 10^−6^ g/L)	m	AEC/SWV	[173]
cap: SH-(CH_2_)_6_	apt: AGC AGC ACA GAG GTC AGA TGA CTT CAG TGA GTT GTC CCA CGG TCG GCG AGT CGG TGG TAG CCT ATG CGT GCT ACC GTG AA cap: GAG GAT TCA GTG A cDNA I: CCG ACC GTG GGA CAA CTC AGT GAA cDNA II: ACG GTC GGT TAC A	cDNA II: biotin	-	5 (b)	m	AEC/SWV	[41]
apt: NH_2_- (CH_2_)_6_ cDNA: NH_2_-(CH_2_)_6_	apt: ACT TCA GTG AGT TGT CCC ACG GTC GGC GAG TCG GTG GTA G cDNA: ACC GAC TCG CCG ACC	-	-	0.00019 (b)	m	AEC/SWV	[49]
cDNA I: NH_2_-(CH_2_)_6_ cDNA II: SH-(CH_2_)_6_	apt: ACT TCA GTG AGT TGT CCCACG GTC GGC GAG TCG GTG GTA GCC TAT GCA GTT T cDNA I: TTT CGC TGT GAC CTA CCA CCG ACT GC cDNA II: TTT GTG CAT AGG GTC ACA G	-	-	0.0000033 (b)	m	AEC/SWV	[174]
SH-(CH_2_)_6_	ACT TCA GTG AGT TGT CCC ACG GTC GGC GAG TCG GTG GTA G	-	-	2.0 (b)	m	AEC/LSV	[175]
apt: SH	apt: AGC AGC ACA GAG GTC AGA TGA CTG AGG GCA CGG ACA GGA GGG CAT GGA GAG ATG GCG		-	0.000021 (b)	m	AEC /SWV	[112,150]

^1^ When naming several references, the first always describes the realized sensor with associated LOD; aptamer sequence(s) and/or associated K_D_ values are derived from the additional reference(s).

**Table 10:** Aptamer sequence, dissociation constant (K_D_), limit of detection (LOD), real sample analysis (RSA), and realized sensor type and measuring method for ciprofloxacin, mentioned in the corresponding references (Ref). AEC = amperometric electrochemical, b = buffer, cDNA = complementary DNA, CO = colorimetric, DPV = differential pulse voltammetry, hs = human serum, m = milk, and sw = spiked water.

5′ Linker and Spacer	Aptamer Sequence 5′→3′	3′ Linker and Spacer	K_D_ (nM)	LOD (nM)	RSA	Sensor Type/Method	Ref ^1^
cDNA II: SH	apt: ATA CCA GCT TAT TCA ATT GCA GGG TAT CTG AGG CTT GAT CTA CTA AAT GTC GTG GGG CAT TGC TAT TGG CGT TGA TAC GTA CAA TCG TAA TCA GTT AG cDNA I: TTG AAT AAG CTG GTA TAA ACC cDNA II: AAA CCA CCT CCG AAT CCC AAG CCA CCG CCG CTA ACT GAT TAC GAT TGT	cDNA I: SH	-	1.3 (sw) 2.6 (s) 3.2 (m)	sw, hs, m	CO	[179,185]
SH	ATA CCA GCT TAT TCA ATT GCA GGG TAT CTG AGG CTT GAT CTA CTA AAT GTC GTG GGG CAT TGC TAT TGG CGT TGA TAC GTA CAA TCG TAA TCA GTT AG	-	-	0.263 (b)	m, hs	AEC/DPV	[180]

^1^ When naming several references, the first always describes the realized sensor with associated LOD; aptamer sequence(s) and/or associated K_D_ values are derived from the additional reference(s).

**Table 11:** Aptamer sequence, dissociation constant (K_D_), limit of detection (LOD), real sample analysis (RSA), and realized sensor type and measuring method for danofloxacin, mentioned in the corresponding references (Ref). FAM = fluorescein amidite, FL = fluorometric, and SPR = surface plasmon resonance.

5′ Linker and Spacer	Aptamer Sequence 5′→3′	3′ Linker and Spacer	K_D_ (nM)	LOD (nM)	RSA	Sensor Type/Method	Ref ^1^
FAM-oligo(dT)	UCA GGC UCC UGU GAA GCA ACC GAA UGG ACU GA	A_16_	1.81 ± 0.18	-	-	FL, SPR	[181]

^1^ When naming several references, the first always describes the realized sensor with associated LOD; aptamer sequence(s) and/or associated K_D_ values are derived from the additional reference(s).

**Table 12:** Aptamer sequence, dissociation constant (K_D_), limit of detection (LOD), real sample analysis (RSA), and realized sensor type and measuring method for enrofloxacin, mentioned in the corresponding references (Ref). apt = aptamer, b = buffer, cDNA = complementary DNA, and FL = fluorometric.

5′ Linker and Spacer	Aptamer Sequence 5′→3′	3′ Linker and Spacer	K_D_ (nM)	LOD (nM)	RSA	Sensor Type/Method	Ref ^1^
-	apt: CCC ATC AGG GGG CTA GGC TAA CAC GGT TCG GCT CTC TGA GCC CGG GTT ATT TCA GGG GGA cDNA: GTG TTA GCC TAG CCC CCT GAT	apt: biotin cDNA: biotin	-	0.56 (b) (0.02 × 10^−6^ g/L)	f	FL	[182]
-	CCC ATC AGG GGG CTA GGC TAA CAC GGT TCG GCT CTC TGA GCC CGG GTT ATT TCA GGG GGA	biotin	-	0.11 (b) (0.04 × 10^−6^ g/L)	f	FL	[183]

^1^ When naming several references, the first always describes the realized sensor with associated LOD; aptamer sequence(s) and/or associated K_D_ values are derived from the additional reference(s).

**Table 13:** Aptamer sequence, dissociation constant (K_D_), limit of detection (LOD), real sample analysis (RSA), and realized sensor type and measuring method for oflofloxacin, mentioned in the corresponding references (Ref). AEC = amperometric electrochemical, b = buffer, CV = cyclic voltammetry, DPV = differential pulse voltammetry, FL = fluorometric, p = pork, and tp = tap water.

5′ Linker and Spacer	Aptamer Sequence 5′→3′	3′ Linker and Spacer	K_D_ (nM)	LOD (nM)	RSA	Sensor Type/Method	Ref ^1^
SH-(CH_2_)_6_	ATA CCA GCT TAT TCA ATT AGT TGT GTA TTG AGG TTT GAT CTA GGC ATA GTC AAC AGA GCA CGA TCG ATC TGG CTT GTT CTA CAA TCG TAA TCA GTT AG	-	0.2	1.0 (b)	p, tp	AEC/CV, DPV	[184,185]
-	apt I: ATA CCA GCT TAT TCA ATT CGA TGG TAA GTG AGG TTC GTC CCT TTA ATA AAC TCG ATT AGG ATC TCG TGA GGT GTG CTC TAC AAT CGT AAT CAG TTA G apt II: ATA CCA GCT TAT TCA ATT GCA GGG TAT CTG AGG CTT GAT CTA CTA AAT GTC GTG GGG CAT TGC TAT TGG CGT TGA TAC GTA CAA TCG TAA TCA GTT AG apt III: ATA CCA GCT TAT TCA ATT AGT TGT GTA TTG AGG TTT GAT CTA GGC ATA GTC AAC AGA GCA CGA TCG ATC TGG CTT GTT CTA CAA TCG TAA TCA GTT AG	-	I: 56.9 ± 11.3 II: 0.11 ± 0.06 III: 0.20 ± 0.09	-	-	FL	[185]

^1^ When naming several references, the first always describes the realized sensor with associated LOD; aptamer sequence(s) and/or associated K_D_ values are derived from the additional reference(s).

**Table 14:** Aptamer sequence, dissociation constant (K_D_), limit of detection (LOD), real sample analysis (RSA) and realized sensor type and measuring method for lincomycin, mentioned in the corresponding references (Ref). AC = alternating current, b = buffer, CV = cyclic voltammetry, ECL = electrochemiluminescent, and me = meat.

5′ Linker and Spacer	Aptamer Sequence 5′→3′	3′ Linker and Spacer	K_D_ (nM)	LOD (nM)	RSA	Sensor Type/Method	Ref ^1^
C dot	CGC GTG ATG TGG TCG ATG CGA TAC GGT GAG TCG CGC CAC GGC TAC ACA CGT CTC AGC GA	-	-	0.00016 (b)	me	ECL/CV, AC	[186]

^1^ When naming several references, the first always describes the realized sensor with associated LOD; aptamer sequence(s) and/or associated K_D_ values are derived from the additional reference(s).

**Table 15:** Aptamer sequence, dissociation constant (K_D_), limit of detection (LOD), real sample analysis (RSA), and realized sensor type and measuring method for oxytetracycline, mentioned in the corresponding references (Ref). AEC = amperometric electrochemical, apt = aptamer, b = buffer, CAN = cantilever, cDNA = complementary DNA, CO = colorimetric, CV = cyclic voltammetry, d = drugs, DPV = differential pulse voltammetry, ECL = electrochemiluminescent, EIS = electrochemical impedance spectrometry, ELAA = enzyme-linked aptamer assay, f = fish, FAM = fluorescein amidite, FL = fluorometric, h = honey, LSPIA = light scattering particle immunoagglutination assay, lw = lake water, m = milk, mb = mouse blood, ms = mouse serum, mu = mouse urine, p = pork, PEC = photoelectrochemical, rw = river water, SERS = surface-enhanced Raman scattering, SWV = square wave voltammetry, and tw = tap water.

5′ Linker and Spacer	Aptamer Sequence 5′→3′	3′ Linker and Spacer	K_D_ (nM)	LOD (nM)	RSA	Sensor Type/Method	Ref ^1^
-	I: CGT ACG GAA TTC GCT AGC CGA CGC GCG TTG GTG GTG GAT GGT GTG TTA CAC GTG TTG TGG ATC CGA GCT CCA CGT G II: CGT ACG GAA TTC GCT AGC ACG TTG ACG CTG GTG CCC GGT TGT GGT GCG AGT GTT GTG T GG ATC CGA GCT CCA CGT G III: CGT ACG GAA TTC GCT AGC CGA GTT GAG CCG GGC GCG GTA CGG GTA CTG GTA TGT GTG G GG ATC CGA GCT CCA CGT G	-	I: 9.61 II: 12.08 III: 56.84	-	-	FL	[187,194]
-	AGG TGC AC	-	1.104	0.1 (b)	-	CO/UV–VIS	[195]
-	GGA ATT CGC TAG CAC GTT GAC GCT GGT GCC CGG TTG TGG TGC GAG TGT TGT GTG GAT CCG AGC TCC ACG TG	(CH_2_)_6_-SH		0.2 (b)	-	CAN	[187,197]
-	CGA ACG CGC GTT GGT GGT GGA TGG TGT GTT ACA CGT GTT GT	-	9.61	100 (b)	-	LSPIA	[187,189]
-	I: CGA CGC ACA GTC GCT GGT GCG TAC CTG GTT GCC GTT GTG T II: GGC GCG GCA TGG TGT GGA CTC CAG GCG GTA GGG ATG TCG T III: GGC GAA GGA GTC ATG TAG GTG TGG TCG AGA CCG CTG TGC T IV: GAA AGG GAC GTT CCA AGT TCG TAT AAG CAG TCC TGT GCG T	-	I: 4.7 II: 8.0 III: 9.5 IV: 14.0	I: 26.7 (b) (12.3 × 10^−6^ g/L) I: 58.6 (m) (27 × 10^−6^ g/L)	m	ELAA	[192]
biotin	ACC GCA CCA CCG TCA TGA GTG CGA ACT TAC GCA ATC ATG ACG GTG GTG CGG TGG TG	SH	-	0.000000009 (b) (0.0435 × 10^−12^ g/L)	f	SERS	[195,196]
-	CGT ACG GAA TTC GCT AGC GGG CGG GGG TGC TGG GGG AAT GGA GTG CTG CGT GCT GCG GGG ATC CGA GCT CCA CGT G	-	11.13	25 (b)	-	CO/UV–VIS	[187,190]
-	CGT ACG GAA TTC GCT AGC GGG CGG GGG TGC TGG GGG AAT GGA GTG CTG CGT GCT GCG GGG ATC CGA GCT CCA CGT G	-	-	1 (b) 1 (tw)	tw	CO/UV–VIS	[187,198]
FAM	CGT ACG GAA TTC GCT AGC GGG CGG GGG TGC GGG AAT GGA GTG CTG CGT GCT GCG GGG ATC CGA GCT CCA CGT G	-		10 (b)	lw	FL	[34,187]
apt: biotin cDNA: FAM	apt: GGA ATT CGC TAG CAC GTT GAC GCT GGT GCC CGG TTG TGG TGC GAG TGT TGT GTG GAT CCG AGC TCC ACG TG cDNA: ACA CAA CAC TCG CAC CAC AAC CGG GCA CCA GCG TCA ACG T	-	-	1.85 (b) (0.85 × 10^−6^ g/L)	m, h, p	FL	[82,187]
-	apt: CGT ACG GAA TTC GCT AGC GGG CGG GGG TGC GGG AAT GGA GTG CTG CGT GCT GCG GGG ATC CGA GCT CCA CGT G cDNA I: AAT TCC GTA CG cDNA II: CGT ACG GAA TT	cDNA I: FAM	-	10 (b)	m, tw	FL	[199]
FAM	CGT ACG GAA TTC GCT AGC GGG CGG GGG TGC TGG GGG AAT GGA GTG CTG CGT GCT GCG GGG ATC CGA GCT CCA CGT G	-	-	54.3 (b) (25 × 10^−6^ g/L)	tw, rw	FL	[200]
apt: NH_2_ cDNA: NH_2_	apt: GGA ATT CGC TAG CAC GTT GAC GCT GGT GCC CGG TTG TGG TGC GAG TGT TGT GTG GAT CCG AGC TCC ACG TG cDNA: CGG ATC CAC ACA ACA	-	-	0.078 (b) (0.036 × 10^−6^ g/L)	m	ECL	[40,187]
apt: NH_2_ cDNA: NH_2_	apt: GGA ATT CGC TAG CAC GTT GAC GCT GGT GCC CGG TTG TGG TGC GAG TGT TGT GTG GAT CCG AGC TCC ACG TG cDNA: CAA CGT GCT AGC GAA	-	-	0.12 (b) (0.054 × 10^−6^ g/L)	m	ECL	[187,201]
apt: biotin cDNA: SH-(CH_2_)_6_	apt: GGA ATT CGC TAG CAC GTT GAC GCT GGT GCC CGG TTG TGG TGC GAG TGT GTG GAT CCG AGC TCC ACG TG cDNA: AAA ATC CAC ACA ACA	-	-	0.043 (b) (0.02 × 10^−6^ g/L)	m	ECL	[93]
NH_2_-(CH_2_)_6_	GGA ATT CGC TAG CAC GTT GAC GCT GGT GCC CGG TTG TGG TGC GAG TGT TGT GTG GAT CCG AGC TCC ACG TG	-	-	0.9 (b)	d	PEC/EIS	[187,202]
cDNA I: SH cDNA II: SH cDNA III: SH	apt: GGA ATT CGC TAG CAC GTT GAC GCT GGT GCC CGG TTG TGG TGC GAG TGT TGT GTG GAT CCG AGC TCC ACG TG cDNA I: CAC GTG GAG CTC GGA TCC ACA CAA CAC TCG CAC CAC AAC CGG GCA CCA GCG TCA ACG TGC TAG CGA ATT CC cDNA II: CAC GTG GAG CTC GGA TCC AC cDNA III: CAC GTG GAG CTC GGA TCC ACA CAA CAC TCG CAC CA	cDNA I: (TTT)_20_-ACG TG-NH_2_ cDNA II: (TTT)_5_-ACG TG-NH_2_ cDNA III: (TTT)_10_-ACG TG-NH_2_	-	0.19 (b)	m, w, c	PEC	[203]
-	GGA ATT CGC TAG CAC GTT GAC GCT GGT GCC CGG TTG TGG TGC GAG TGT TGT GTG GAT CCG AGC TCC ACG TG	C_3_-SH	11.13	1 (b)	-	AEC/SWV	[187,188]
cDNA: SH-(CH_2_)_6_	apt: CGT ACG GAA TTC GCT AGC GGG CGG GGG TGC GGG AAT GGA GTG CTG CGT GCT GCG GGG ATC CGA GCT CCA CGT G cDNA: GCA TGC CTT AAG CGA TCG CCA TAT TAT AAG GCA TGC	cDNA: ferrocene	-	21.3 (b) (9.8 × 10^−6^ g/L)	mb, ms, mu	AEC/SWV	[204]
biotin C_3_	GGA ATT CGC TAG CAC GTT GAC GCT GGT GCC CGG TTG TGG TGC GAG TGT TGT	-	-	0.005 (b) (2.3 × 10^−9^ g/L)	h	AEC/CV	[205]
-	TCA CGT TGA CGC TGG TGC CCG GTT GTG GTG GGA GTG TTG TGT	(CH_2_)_6_-NH_2_	4.7	0.00018 (b)	m	AEC/SWV	[107,187]
cDNA I: SH-(CH_2_)_6_ cDNA II: NH_2_-(CH_2_)_6_	apt: ACG TTG ACG CTG GTG CCC GGT TGT GGT GGG AGT GTT GTG T cDNA I: CTA CCA TTT TTT CGC CGA CC cDNA II: GGT CGG CGA AAA AAT GGT AG	cDNA I: (CH_2_)_6_-PHO	-	0.22 (b) (0.1 × 10^−6^ g/L)	m	AEC/SWV	[173]
cDNA I: NH_2_-(CH_2_)_6_ cDNA II: SH-(CH_2_)_6_	apt: ACG TTG ACG CTG GTG CCC GGT TGT GGT GCG AGT GTT GTG TCC TAT GCA GTT T cDNA I: TTT CGC TGT GAC ACA CAA CAC TCG GT cDNA II: TTT GTG CAT AGG GTC ACAG	-	-	0.0000048 (b)	m	AEC/SWV	[174]
SH	CGA CGC ACA GTC GCT GGT GCG TAC CTG GTT GCC GTT GTG T	-	-	0.498 (b)	h	AEC/DPV	[206]

^1^ When naming several references, the first always describes the realized sensor with associated LOD; aptamer sequence(s) and/or associated K_D_ values are derived from the additional reference(s).

**Table 16:** Aptamer sequence, dissociation constant (K_D_), limit of detection (LOD), real sample analysis (RSA), and realized sensor type and measuring method for tetracycline, mentioned in the corresponding references (Ref). AEC = amperometric electrochemical, apt = aptamer, b = buffer, cap = capture probe, cDNA = complementary DNA, CO = colorimetric, DPV = differential pulse voltammetry, EIS = electrochemical impedance spectrometry, ELAA = enzyme-linked aptamer assay, ESI-MS = electrospray ionization-ion mobility spectrometry, FAM = fluorescein amidite, FIS = Faradaic impedance spectroscopy, FL = fluorometric, h = honey, hp = human plasma, hs = human serum, hu = human urine, IEC = impedimetric electrochemical, m = milk, p = pork, PEC = photoelectrochemical, rs = rat serum, SERS = surface enhanced Raman scattering, SWV = square wave voltammetry, tw = tap water, u = urine, and uw = ultrapure water.

5′ Linker and Spacer	Aptamer Sequence 5′→3	3′ Linker and Spacer	K_D_ (nM)	LOD (nM)	RSA	Sensor Type/Method	Ref ^1^
-	I: CGT ACG GAA TTC GCT AGC CCC CCG GCA GGC CAC GGC TTG GGT TGG TCC CAC TGC GCG TGG ATC CGA GCT CCA CGT G II: CGT ACG GAA TTC GCT AGC GGG GGC ACA CAT GTA GGT GCT GTC CAG GTG TGG TTG TGG TGG ATC CGA GCT CCA CGT G III: CGT ACG GAA TTC GCT AGC GGG CGG GGG TGC TGG GGG AAT GGA GTG CTG CGT GCT GCG G GG ATC CGA GCT CCA CGT G	-	I 63 II 70 III 100	-	-	-	[194]
NH_2_	CGT ACG GAA TTC GCT AGC CCC CCG GCA GGC CAC GGC TTG GGT TGG TCC CAC TGC GCG TGG ATC CGA GCT CCA CGT G	C_6_	63.6	42.8 (hu) (0.019 × 10^−3^ g/L) 83.3 (p) (0.037 × 10^−3^ g/L)	hu, hp	ESI-IMS	[207,228]
II biotin	I CGT ACG GAA TTC GCT AGC CCC CCG GCA GGC CAC GGC TTG GGT TGG TCC CAC TGC GCG TGG ATC CGA GCT CCA CGT G II GAG CCU AAA ACA UAC CAG AGA AAU CUG GAG AGG UGA AGA AUA CGA CCA CCU AGG CUC	I biotin	I: 63 II: 0.77	I: 32.7 (b) I: 95.2 (m) II: 21.0 (b) II: 35.1 (m)	m	ELAA	[194,209,210]
-	CGT ACG GAA TTC GCT AGC CCC CCG GCA GGC CAC GGC TTG GGT TGG TCC CAC TGC GCG TGG ATC CGA GCT CCA CGT G	biotin	63.6	0.018 (b) (7.8 × 10^−9^ g/L) 0.022 (h) (9.6 × 10^−9^ g/L)	h	ELAA	[194,240]
-	CGT ACG GAA TTC GCT AGC CCC CCG GCA GGC CAC GGC TTG GGT TGG TCC CAC TGC GCG TGG ATC CGA GCT CCA CGT G	biotin	-	0.15 (b) (0.0659 × 10^−6^ g/L) 0.22 (h) (0.0978 × 10^−6^ g/L)	h	ELAA	[194,241]
-	CGT ACG GAA TTC GCT AGC CCC CCG GCA GGC CAC GGC TTG GGT TGG TCC CAC TGC GCG TGG ATC CGA GCT CCA CGT G	-	-	11.6 (b)	m	SERS	[194,216]
apt: NH_2_ cDNA: NH_2_	apt: CGT ACG GAA TTC GCT AGC CCC CCG GCA GGC CAC GGC TTG GGT TGG TCC CAC TGC GCG TGG ATC CGA GCT CCA CGT G cDNA: CAA CGT GCT AGC GAA	apt: NH_2_	-	0.0023 (b) (0.001 × 10^−6^ g/L)	m	SERS	[217]
-	AGG TGC AC	-	1.067	0.1 (b)	-	CO/UV–VIS	[195]
-	CGT ACG GAA TTC GCT AGC CCC CCG GCA GGC CAC GGC TTG GGT TGG TCC CAC TGC GCG TGG ATC CGA GCT CCA CGT G	-	-	122 (b)	m	CO/UV–VIS	[194,211]
-	CGT ACG GAA TTC GCT AGC CCC CCG GCA GGC CAC GGC TTG GGT TGG TCC CAC TGC GCG TGG ATC CGA GCT CCA CGT G	-	-	45.8 (uw)	m	CO/UV–VIS	[194,212]
-	CGT ACG GAA TTC GCT AGC CCC CCG GCA GGC CAC GGC TTG GGT TGG TCC CAC TGC GCG TGG ATC CGA GCT CCA CGT G	-	63.6	87.8 (b) (0.039 × 10^−3^ g/L)	m	CO/UV–VIS	[194,242]
-	CTC TCT CGG TGG TGT CTC TC	-	-	0.266 (b) 0.347 (m) 0.393 (rs)	m, rs	CO/UV–VIS	[195,213]
-	CGT ACG GAA TTC GCT AGC CCC CCG GCA GGC CAC GGC TTG GGT TGG TCC CAC TGC GCG TGG ATC CGA GCT CCA CGT G	-	3.4	-	-	CO/UV–VIS	[194,195,214]
-	CGT ACG GAA TTC GCT AGC CCC CCG GCA GGC CAC GGC TTG GGT TGG TCC CAC TGC GCG TGG ATC CGA GCT CCA CGT G	-	-	0.0023 (b) (0.001 × 10^−6^ g/L)	h	CO/UV–VIS	[215]
I: (CT)_4_ II: (CT)_4_C II: (CT)_5_ cDNA: FAM	I, II, III: GGG GGC ACA CAT GTA GGT GCT GTC CAG GTG TGG TTG TGG T cDNA: GAG GAG AGA GAG AGA TCC TC	I: (TC)_3_ II: (CT)_3_C III: (TC)_4_ cDNA: black hole quencher		I: 2.09 (b) I: 7.30 (tw) I: 8.48 (rs)	tw, rs	FL	[218]
-	apt: TCC CTT CCG GTG GTG CTT CCC T G-quadruplex: ATG GGA AGG GAG GGA TGG GT	-	-	0.97 (b)	hs	FL	[195,219]
-	apt: CGT ACG GAA TTC GCT AGC CCC CCG GCA GGC CAC GGC TTG GGT TGG TCC CAC TGC GCG TGG ATC CGA GCT CCA CGT G cDNA: CAA CGT GCT AGC GAA	-	-	0.014 (b) (6.2 × 10^−9^ g/L)	m, p	FL	[194,220]
-	CGT ACG GAA TTC GCT AGC CCC CCG GCA GGC CAC GGC TTG GGT TGG TCC CAC TGC GCG TGG ATC CGA GCT CCA CGT G	-	-	0.65 (uw) (0.29 × 10^−6^ g/L)	m	FL	[194,221]
apt: biotin cDNA: SH-(CH_2_)_6_	apt: CGT ACG GAA TTC GCT AGC CCC CCG GCA GGC CAC GGC TTG GGT TGG TCC CAC TGC GCG TGG ATC CGA GCT CCA CGT G cDNA: GGA CCA ACC CAA	-	-	0.045 (b) (0.02 × 10^−6^ g/L)	m	ECL	[93]
-	CGT ACG GAA TTC GCT AGC CCC CCG GCA GGC CAC GGC TTG GGT TGG TCC CAC TGC GCG TGG ATC CGA GCT CCA CGT G	-	-	5.3 (b)	lw	PEC	[208,242]
SH-(CH_2_)_6_	CGT ACG GAA TTC GCT AGC CCC CCG GCA GGC CAC GGC TTG GGT TGG TCC CAC TGC GCG TGG ATC CGA GCT CCA CGT G	-	-	0.1 (b)	m	PEC	[222,241]
-	CGT ACG GAA TTC GCT AGC CCC CCG GCA GGC CAC GGC TTG GGT TGG TCC CAC TGC GCG TGG ATC CGA GCT CCA CGT G		-	4.5 (b)	w	PEC	[223,242]
-	ACT CTT ATA CGG GAG CCA ACA CCA AAG CTT CTG CGC CAC ACC ATA TGA GAG CAG GTG GTA CGG ATA AGC T	-	52.5 ± 3.6	22.5 (b) (1 × 10^−5^ g/L)	m	IEC/EIS	[224]
SH-(CH_2_)_6_	GTC TCT GTG TGC GCC AGA GAA CAC TGG GGC AGA TAT GGG CCA GCA CAG AAT GAG GCC C	-	-	6.75 (b) (3.0 × 10^−6^ g/L)	m	IEC/EIS	[147,225]
SH-(CH_2_)_6_	GTC TCT GTG TGC GCC AGA GAA CAC TGG GGC AGA TAT GGG CCA GCA CAG AAT GAG GCC C	-	-	1.0 (b)	m	IEC/EIS	[147,226]
NH_2_-CH_2_	CGT ACG GAA TTC GCT AGC CCC CCG GCA GGC CAC GGC TTG GGT TGG TCC CAC TGC GCG TGG ATC CGA GCT CCA CGT G	-	-	1.0 (b)	m	IEC/EIS	[227]
biotin-T_5_	TTT TTG GTA CGG AAT TCG CTA GCC CCC CHG CAG GCC ACG GCT TGG GTT GGT CCC ACT GCG CGT GGA TCC GAG CTC CAC GTG	-	63.6	10 (b)	-	AEC/SWV	[194,228]
-	-	-	-	2.25 (b) (1.0 × 10^−6^ g/L)	m	AEC/CV	[229]
NH_2_-CH_2_	CGT ACG GAA TTC GCT AGC CCC CCG GCA GGC CAC GGC TTG GGT TGG TCC CAC TGC GCG TGG ATC CGA GCT CCA CGT G	-	-	5.0 (b)	m	AEC/DPV	[194,230]
NH_2_-(CH_2_)_6_	-	-	51800	2.25 (b) (1.0 × 10^−6^ g/L)	m	AEC/DPV	[231]
NH_2_-CH_2_	CGT ACG GAA TTC GCT AGC CCC CCG GCA GGC CAC GGC TTG GGT TGG TCC CAC TGC GCG TGG ATC CGA GCT CCA CGT G	-	-	0.32 (b)	m	AEC/DPV	[194,232]
NH_2_-CH_2_	CGT ACG GAA TTC GCT AGC CCC CCG GCA GGC CAC GGC TTG GGT TGG TCC CAC TGC GCG TGG ATC CGA GCT CCA CGT G	-	-	0.0042 (b)	m	AEC/CV IEC/EIS	[194,233]
NH_2_	CGT ACG GAA TTC GCT AGC CCC CCG GCA GGC CAC GGC TTG GGT TGG TCC CAC TGC GCG TGG ATC CGA GCT CCA CGT G	-	-	0.000029 (b)	d	AEC/DPV	[234]
SH-(CH_2_)_6_	GTC TCT GTG TGC GCC AGA GAA CAC TGG GGC AGA TAT GGG CCA GCA CAG AAT GAG GCC C	-		10 (b)	m	AEC/CV	[147,235]
NH_2_	CGT ACG GAA TTC GCT AGC CCC CCG GCA GGC CAC GGC TTG GGT TGG TCC CAC TGC GCG TGG ATC CGA GCT CCA CGT G	-		aptasensor I: 0.0003 (b, EIS) 0.029 (b, DPV) aptasensor II: 0.0000038 (b, EIS) 0.00031 (b, DPV)	m, d, h, bs	AEC/DPV IEC/EIS	[236]
cDNA I: SH cDNA II: SH	apt: CGT ACG GAA TTC GCT AGC CCC CCG GCA GGC CAC GGC TTG GGT TGG TCC CAC TGC GCG TGG ATC CGA GCT CCA CGT G cDNA I: CCA TCA GAC CTA CCA AAC ACG TGG AGC T cDNA II: AGA CCT ACC AAA CGA ACC CA cDNA III: AAT TCC GTA CGA AAC CAT CCA GAC TAC C	cDNA III: SH	63	0.45 (b) 0.74 (m) 0.71 (s)	m, hs	AEC/DPV	[194,237]
cap: SH-(CH_2_)_6_	apt: CGT ACG GAA TTC GCT AGC CCC CCG GCA GGC CAC GGC TTG GGT TGG TCC CAC TGC GCG TGG ATC CGA GCT CCA CGT G cap: ATG TAG CTA GGT G cDNA I: CGT GTA GCA CAG CAT CAC CAC CTA GC cDNA II: GCT ACA CGC GTT T	cS II: biotin	-	20 (b)	m	AEC/SWV	[41]
-	CGT ACG GAA TTC GCT AGC CCC CCG GCA GGC CAC GGC TTG GGT TGG TCC CAC TGC GCG TGG ATC CGA GCT CCA CGT G	-	-	0.6 (b)	-	AEC/DPV	[238]
SH-(CH_2_)_6_	CGT ACG GAA TTC GCT AGC CCC CCG GCA GGC CAC GGC TTG GGT TGG TCC CAC TGC GCG TGG ATC CGA GCT CCA CGT G	-	-	0.74 (b) (0.33 × 10^−6^ g/L)	m	AEC/DPV	[147,239]

^1^ When naming several references, the first always describes the realized sensor with associated LOD; aptamer sequence(s) and/or associated K_D_ values are derived from the additional reference(s).

**Table 17:** Aptamer sequence, dissociation constant (K_D_), limit of detection (LOD), real sample analysis (RSA), and realized sensor type and measuring method for sulfadimethoxine, mentioned in the corresponding references (Ref). apt = aptamer, b = buffer, cDNA = complementary DNA, CO = colorimetric, d = drugs, f = fish, FAM = fluorescein amidite, FL = fluorometric, lw = lake water, m = milk, and PEC = photoelectrochemical.

5′ Linker and Spacer	Aptamer Sequence 5′→3′	3′ Linker and Spacer	K_D_ (nM)	LOD (nM)	RSA	Sensor Type/Method	Ref ^1^
-	I: GTT AGA TGG GAG GTC ATA TAG C II: GAG GGC AAC GAG TGT TTA TAG A	-	I: 150 II: 84	II: 32.2 (b) II: 32.2 (m) (10 × 10^−6^ g/L)	m	FL	[243]
-	GAG GGC AAC GAG TGT TTA TAG A	FAM	-	-	-	FL, CO/UV–VIS	[243,246]
-	GAG GGC AAC GAG TGT TTA TAG A	-	-	161.1 (b) (50 × 10^−6^ g/L)	-	CO/UV–VIS	[243,244]
-	GAG GGC AAC GAG TGT TTA TAG A	-	84	-	-	CO/UV–VIS	[75,243]
-	GAG GGC AAC GAG TGT TTA TAG A	-	-	32.2 (b) (10 × 10^−6^ g/L)	m	CO/UV–VIS	[246]
-	GAG GGC AAC GAG TGT TTA TAG A	-	-	22.56 (b)	lw	CO	[247]
-	apt: GAG GGC AAC GAG TGT TTA TAG A cDNA: CGT TGC CCT C	apt: biotin cDNA: biotin	-	0.35 (b) (0.11 × 10^−6^ g/L)	f	FL	[243,248]
NH_2_	GAG GGC AAC GAG TGT TTA TAG A	FAM	-	0.55 (b)	m, d	PEC	[249]

^1^ When naming several references, the first always describes the realized sensor with associated LOD; aptamer sequence(s) and/or associated K_D_ values are derived from the additional reference(s).

## Abbreviations

AC	alternating current
AEC	amperometric electrochemical
AuNPs	gold nanoparticles
bsa	bovine serum albumin
CA	chronoamperometry
CAN	cantilever
cDNA	complementary DNA
CNT	carbon nanotube
CO	colorimetric
CRET	chemiluminescence resonance energy transfer
CSRP	circular strand-replacement DNA polymerization
CV	cyclic voltammetry
Cy3/Cy5	cyanine 3, cyanine 5
dsDNA	double stranded DNA
DPV	differential pulse voltammetry
EBFC	enzyme biofuel cell
ECL	electrochemiluminescent
EIS	electrochemical impedance spectroscopy
ELAA	enzyme-linked aptamer assay
ELISA	enzyme-linked immunosorbent assay
ESI-IMS	electrospray ionization-ion mobility spectrometry
FAM	fluorescein amidite
FIS	faradaic impedance spectroscopy
FL	fluorometric
FRET	fluorescence resonance energy transfer
GO	graphene oxide
HRP	horseradish peroxidase
IEC	impedimetric electrochemical
K_D_	dissociation constant
LOD	limit of detection
LCA	liquid crystal assay
LSPIA	light scattering particle immunoagglutination assay
LSV	linear sweep voltammetry
MB	methylene blue
MCH	6-mercapto-1-hexanol
MIP	molecularly imprinted polymer
MOF	metal organic framework
MWCNTs	multi-walled carbon nanotubes
NPs	nanoparticles
OCV	open circuit voltage
PEC	photoelectrochemical
QCM	quartz crystal microbalance
QD	quantum dot
rGO	reduced graphene oxide
ROX	carboxy-x-rhodamine
SAM	self-assembled monolayer
SAW	surface acoustic wave
SELEX	systematic evolution of ligands by exponential enrichment
SERS	surface-enhanced Raman scattering
SPCE	screen-printed carbon electrode
SPE	screen-printed electrode
SPR	surface plasmon resonance spectroscopy
SSB	ssDNA binding protein
ssDNA	single stranded DNA
SWV	square wave voltammetry
UCNPs	upconversion nanoparticles
